# Spectroscopic Characterization of Radical Pair Photochemistry in Nonmigratory Avian Cryptochromes: Magnetic Field Effects in *Gg*Cry4a

**DOI:** 10.1021/jacs.4c14037

**Published:** 2025-06-30

**Authors:** Jamie Gravell, Patrick D. F. Murton, Tommy L. Pitcher, Kevin B. Henbest, Jessica Schmidt, Madeline M. Buffett, Gabriel Moise, Angela S. Gehrckens, Daniel R. Cubbin, Ana Štuhec, Lewis M. Antill, Olivier Paré-Labrosse, Marco Bassetto, Ghazaleh Saberamoli, Jingjing Xu, Corinna Langebrake, Miriam Liedvogel, Erik Schleicher, Stefan Weber, Rabea Bartölke, Henrik Mouritsen, P. J. Hore, Stuart R. Mackenzie, Christiane R. Timmel

**Affiliations:** † Department of Chemistry, University of Oxford, Chemistry Research Laboratory, Oxford OX1 3TA, U.K.; ‡ Centre for Advanced Electron Spin Resonance (CAESR), Department of Chemistry, 6396 University of Oxford, Oxford OX1 3QR, U.K.; § Department of Chemistry, University of Oxford, Physical and Theoretical Chemistry Laboratory, Oxford OX1 3QZ, U.K.; ∥ AG Neurosensory Sciences/Animal Navigation, Institut für Biologie und Umweltwissenschaften, 11233 Carl-von-Ossietzky Universität Oldenburg, 26129 Oldenburg, Germany; ⊥ 39037 Institute of Avian Research ‘Vogelwarte Helgoland’, 26386 Wilhelmshaven, Germany; # MPRG Behavioural Genomics, MPI Evolutionary Biology, 24306 Plön, Germany; g Institut für Physikalische Chemie, 9174 Albert-Ludwigs-Universität Freiburg, 79104 Freiburg, Germany; h Research Center for Neurosensory Sciences, 11233 Carl-von-Ossietzky Universität Oldenburg, 26111 Oldenburg, Germany

## Abstract

The magnetic compass sensor in night-migratory songbirds is thought to be a flavin-tryptophan radical pair formed by blue-light excitation of the protein cryptochrome-4a (Cry4a) localized in photoreceptor cells in the birds’ retinas. The effects of applied magnetic fields on the photochemistry of purified Cry4a from the migratory European robin are well characterized, but it is less clear what, if anything, distinguishes the magnetic responses of the Cry4a proteins from migratory and nonmigratory species. We present here a detailed study of the magnetic sensitivity of Cry4a from the nonmigratory chicken. The wild-type protein is compared with two mutants in which either Arg317 or Glu320, both close to the tryptophan radical, were replaced by the amino acids Cys and Lys, respectively, found in Cry4a from robins and other night-migratory passerines. These sites had previously been identified as probably facilitating the evolution of an optimized magnetic sensor for nocturnal orientation in songbirds. Neither of these mutations was found to affect the reaction kinetics or magnetic sensitivity of the radical pairs, suggesting that any differences in Cry4a between robin and chicken must stem from their ability to transmit magnetic information, for example via protein–protein interactions. In contrast, a Trp → Phe mutation at the end of the tryptophan-tetrad electron transfer chain in both cryptochromes led to a large increase in magnetic sensitivity, suggesting different sensing and signaling roles for the third and fourth tryptophans.

## Graphical abstract

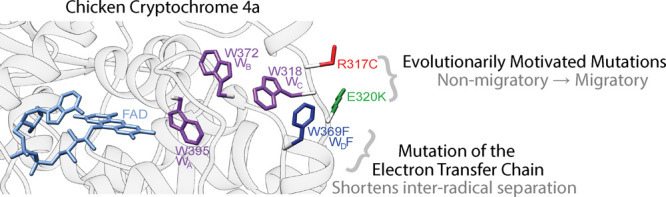

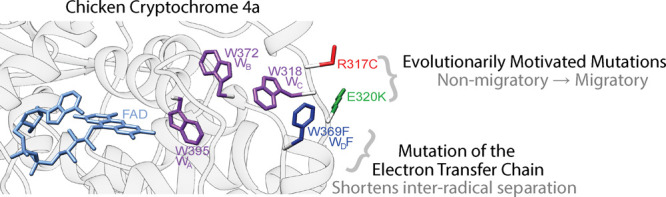

## Introduction

Discovered by Kaptein and Oosterhoff, the radical pair mechanism (RPM) governs the magnetic field-sensitive reaction dynamics of certain chemical reactions, namely those proceeding via short-lived radical pair intermediates.^1^ Crucially, it was proposed by Schulten et al. in 1978 that avian magnetoreception, the ability of certain birds to use the Earth’s magnetic field for their annual migration, might have its origin in RPM-driven, light-induced reactions, now believed to occur within proteins located in the birds’ eyes.^2–7^ More than a decade after Schulten’s original proposal, cryptochromes (Cry) were discovered, first in the model plant (*At*) and later in most kingdoms of life, including animals.^8–11^ As blue-light-sensitive flavoproteins, cryptochromes are closely related both structurally and genetically to their ancestors, the DNA photolyases (PL).^8–11^ Their discovery sparked great interest from zoologists and physical scientists alike, resulting in an array of behavioral animal studies^4,12–17^ and *in vitro* spectroscopic studies on the purified proteins, testing the so-called cryptochrome hypothesis of magnetoreception within the RPM framework.^18–25^

Observations of magnetic field-sensitive, RPM-driven, light-induced reactions in the cryptochrome/photolyase family of proteins, such as *At*Cry1, photolyase (*Ec*PL) and Cry (*Dm*Cry) have added significant weight to the cryptochrome hypothesis.^19–21^ However, it was not until 2021 that the magnetic field sensitivity of a cryptochrome from a migratory animal, the European robin (*Erithacus rubecula, Er*), was demonstrated.^18^ A detailed spectroscopic study of *Er*Cry4a and two Cry4a proteins from nonmigratory species, pigeon (*Columba livia, Cl*) and chicken (*Gallus gallus*, *Gg*), drew several important conclusions.^18^

(i) All three wild-type (WT) avian Cry4a’s form photogenerated spin-correlated radical pairs (SCRPs) via sequential electron transfer between the flavin adenine dinucleotide (FAD) cofactor and tryptophan (W) residues (see Figure 1).
(ii) In *Er*Cry4a WT, electron transfer proceeds along a tetrad of tryptophan residues (which we shall label W_A_, W_B_, W_C_, and W_D_ in order of increasing distance from the FAD cofactor).
(iii) The kinetics and yields of the radical pair reaction in all three proteins are sensitive to applied magnetic fields *in vitro*.
(iv) In *Er*Cry4a, replacement of the fourth tryptophan (W369, W_D_) by a redox-inactive phenylalanine (F) leads to significantly enhanced magnetosensitivity.
(v) The magnetic field sensitivity of *Er*Cry4a WT seems to differ from that observed for *Cl*Cry4a WT and *Gg*Cry4a WT.

**Figure 1:**
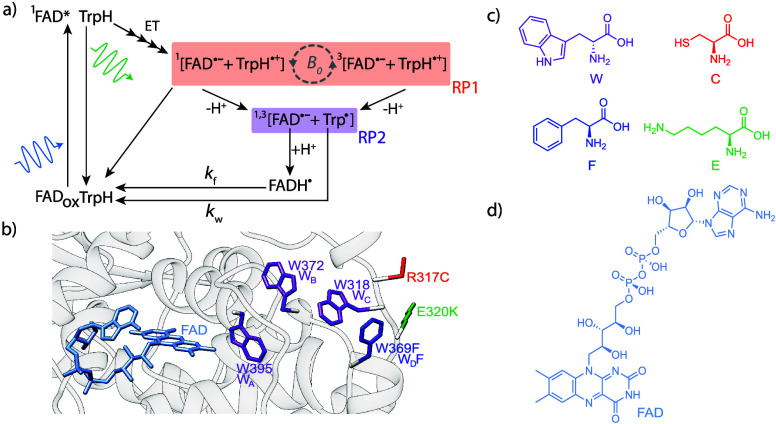
a) Photoreaction scheme proposed for avian cryptochromes. RP1 (orange) formed in an initial singlet state by electron transfer (ET) between FAD (blue in panel b)) and neighboring tryptophan residues (purple in panel b)) can undergo hyperfine driven spin mixing into the triplet state. This mixing is sensitive to an external magnetic field (*B*_0_). Both spin states of RP1 can undergo a deprotonation reaction of the tryptophan radical cation (TrpH^•+^) to yield a radical pair (RP2, purple) which lacks spin correlation. Recombination of RP1 to the ground state by back-electron transfer is only possible from the singlet state. Magnetic field effects on the efficiency of singlet–triplet mixing (gray dashed arrows) thus impact the quantum yield of RP2 and hence FADH^•^ as well as FAD_OX_. The magnetic field effect on the latter is detected by field-sensitive fluorescence (green curly arrow) from the ^1^FAD* excited state. Long-lived FADH^•^ and Trp^•^ radicals return to their ground states by independent oxidation and reduction reactions, respectively. The rate constants for FADH^•^ oxidation and Trp^•^ reduction are *k*_F_ and *k*_W_, respectively. b) Homology model structure (Swiss-model)^29–33^ of *Gg*Cry4a, with residues 317 and 320 exchanged for their counterparts in *Er*Cry4a, R317C and E320K, and residue 369 exchange for phenylalanine, W369F. The model is based on the crystal structure obtained for *Cl*Cry4a, with the C-terminus removed (PDB entry 6PU0).^34^ The moieties involved in the electron transfer chain (FAD and tryptophans) are shown in light blue and purple, respectively. Note that only single point mutants, where the mutation is introduced at either position 317, 320 or 369, were studied in this work. c) Structures of the amino acids tryptophan, phenylalanine, cysteine and lysine. d) Structure of FAD.

Having studied in detail the spectroscopic and magnetic characteristics of *Er*Cry4a and several of its mutants,^18^ the purpose of the work presented here is to provide an equally detailed, and hence, comparative study of a cryptochrome from a nonmigratory bird, namely the chicken. We expressed and studied mutant proteins which provide crucial links between migratory and nonmigratory birds in an attempt to identify any mutations resulting in different spectroscopic, kinetic or magnetic properties. We have also further developed our experimental procedures to allow for efficient recycling of long-lived radicals using the more biologically relevant oxidant, molecular oxygen (see Section SM.3–7 in the Supporting Information) instead of potassium ferricyanide used in previous work.^18,21^

The starting point of any such study must be a close inspection of the different species’ Cry4a sequences, noting any differences involving or neighboring the electron transfer chain.^11^ The tetrad of tryptophans, which provides the electron transfer chain in *Er*Cry4a and *Cl*Cry4a,^18,26^ is conserved widely across avian species, but not in all. Both the robin and pigeon are known from behavioral experiments to use the Earth’s magnetic field.^4,12,13,27,28^ Chickens, by contrast are neither migratory nor homing birds. Herein, we verify the identity of the electron transfer chain that leads to the formation of SCRPs in *Gg*Cry4a.

A detailed phylogenetic analysis of 363 bird genomes, by Langebrake et al., indicated positive selection for cysteine (C) and lysine (K) residues at positions 317 and 320, respectively, in the Cry4a sequence of migratory passerines.^11^ From homology models^18^ of the *Cl*Cry4a crystal structure,^34^ these residues are thought to be in close proximity to the two distal tryptophans, W318 and W369 (W_C_ and W_D_) of the electron transfer chain.^18,26^ Given the apparent importance of both tryptophan residues to the spin chemistry of these proteins (see points (i) to (v) above), a study of the roles of these cysteine and lysine residues in the photochemistry of Cry4a is clearly of interest. To further illustrate the evolutionary conservation of these residues, the amino acid sequences of the Cry4a WT proteins from two night-migratory passerines (*Er*Cry4a and *Sylvia atricapilla, SaC*ry4a), and two nonmigratory, nonpasserine birds (*Gg*Cry4a and *Cl*Cry4a) are provided in the Supporting Information (Figure S2), with the latter two both lacking the conserved residues (C, K) found in the migratory passerines.

Here, we study in detail the effects of single-point mutations of *Gg*Cry4a, with the arginine (R) and glutamic acid (E) residues in positions 317 and 320 exchanged for their respective counterparts from *Er*Cry4a (C and K, respectively). Additionally, we also explore the effect of exchanging the fourth tryptophan residue (W369, W_D_) for a redox inactive phenylalanine (F). Figure 1b-d provides a summary of the mutations, nomenclature, and amino acids discussed in this paper. For this study, we chose to make GgCry4a “more robin-like” (by means of the R317C and E320K mutations) instead of starting with ErCry4a and making it “more chicken-like” (C317R and K320E) because the higher expression yields of the chicken protein allowed a greater range of high-quality measurements than would have been possible with robin Cry4a.

## Results

All the proteins used in this study were expressed and purified using the procedure for *Gg*Cry4a WT in Xu et al.,^18^ except for some improvements made later and described in the Supporting Information (SM.1 and Table S1). The protocol for expressing and purifying *Gg*Cry4a W369F (W_D_F) has been described in Golesworthy et al.^35^ Full details for expression of *Gg*Cry4a R317C and E320K, produced for the first time for this study, are included in the Supporting Information (SM.1 and Table S1). Before any spectroscopic measurements commenced, the diluted protein samples were investigated using native mass spectrometry (SM.2 for experimental details) which confirmed that the proteins were present almost exclusively in their monomeric states and contained the FAD cofactor (Figure S3). Vibrational fine-structure in the UV–visible spectrum in the 300–500 nm region (Figure S4) confirmed that the FAD cofactor was bound within the protein. The absence of any features above 500 nm along with the spectral positions of the vibrational maxima furthermore demonstrated that the proteins contained only the fully oxidized form (FAD_OX_) of the FAD cofactor.

### Electron Paramagnetic Resonance

The first blue-light photosensitive proteins to demonstrate any magnetic field sensitivity were *At*Cry1 and *Ec*PL.^19,20^ These proteins differ from avian Crys in that the electron transfer cascade, which leads to the formation of a magnetically sensitive SCRP (termed RP1), proceeds along a triad, rather than a tetrad, of tryptophan residues.^18,20,24,26,36^ However, the identity of RP1 is the same in all proteins, comprising a flavin semiquinone radical (FAD^•–^) and a tryptophan radical cation (TrpH^•+^) as shown in Figure 1.^18,20,24,37^ Photoinduced electron transfer to FAD in *Dm*Cry, *Er*Cry4a, and *Cl*Cry4a proceeds along a tetrad of tryptophans, extending the electron transfer cascade to W_D_.^18,24,26^ Electron Paramagnetic Resonance (EPR) played the pivotal role in identifying the terminal radical pair in all five proteins. It has been exploited here again to characterize the SCRP in *Gg*Cry4a and its mutants. Experimental and technical details can be found in the Supporting Information (SM.3 and Figure S5).

Briefly, we have applied two EPR techniques common in the study of SCRPs: transient EPR (TrEPR) and out-of-phase electron spin echo envelope modulation (out-of-phase ESEEM).^38–41^ Together, they provide information on radical–radical distances and the identities, spin polarization, and spin correlation of the radicals.

The TrEPR spectra of SCRPs (see SM.3) show distinctive antiphase (e.g., emission–absorption, *e-a*) line-shapes and have been recorded for a number of cryptochromes as well as for *Ec*PL.^18,24,26,42–44^ Additional features contain information on the interaction of the electron spins with the magnetic field, with each other and with nuclear spins in the two radicals.^18,26,42,43,45^ Upon blue-light photoexcitation of *Gg*Cry4a WT, a TrEPR spectrum is obtained whose prevailing antiphase line-shape confirms the formation of a singlet-born SCRP with the fine structure arising from electron–nuclear spin hyperfine couplings (Figure 2a, purple). The spectra obtained for the two mutants, R317C and E320K, are also shown in Figure 2a and, within signal-to-noise, are indistinguishable from that of the WT protein. In contrast, the TrEPR spectrum of the W369F (W_D_F) mutant, lacking the W_D_ residue, is distinctly different (Figure 2b). The antiphase signature, characteristic of a SCRP, is still observed but the splittings are now less well resolved. The different spectrum provides a first indication that mutation of W369 (W_D_) to F interrupts the redox chain significantly, as previously observed for *Dm*Cry W394F, *Er*Cry4a W369F, and *Cl*Cry4a W369F.^18,24,26^

**Figure 2:**
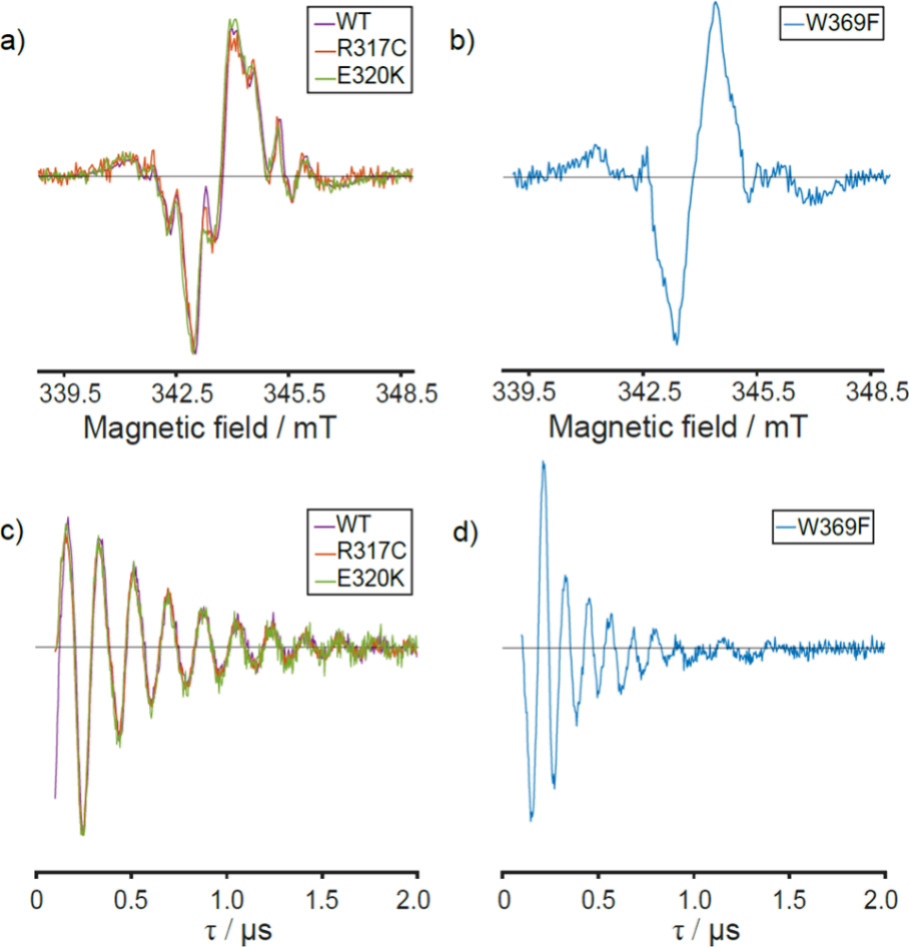
a) X-band (9.75 GHz) TrEPR spectra obtained for *Gg*Cry4a WT (purple), *Gg*Cry4a R317C (red) and *Gg*Cry4a E320K (green) averaged over 0.5–1 μs after a 450 nm laser flash. b) Same as a) for *Gg*Cry4a W369F. The positive and negative signals in all four spectra arise from absorptive (*a*) and emissive (*e*) transitions, respectively. c) Out-of-phase ESEEM traces obtained for *Gg*Cry4a WT (purple), *Gg*Cry4a R317C (red) and *Gg*Cry4a E320K (green) at Q-band frequency (34 GHz). d) Same as c) for *Gg*Cry4a W369F. All EPR data shown here were acquired in the presence of 5 mM K_3_Fe­(CN)_6_, to promote flavin reoxidation. This was found to have a negligible impact on the TrEPR and out-of-phase ESEEM traces obtained (detailed comparisons in the presence and absence of K_3_Fe­(CN)_6_ for both the TrEPR and out-of-phase ESEEM data are shown in Figure S6 and Figure S7, respectively).

We have simulated the TrEPR data (Figure S8), following the treatment outlined in ref 46 and the full set of simulation parameters is provided in the Supporting Information (Table S2). The *g*-factors and hyperfine couplings used for these simulations correspond well with those published elsewhere for FAD and tryptophan radicals.^24,26^ Note that the value of the dipolar coupling, *D*, was fixed during the simulation to the value obtained from the out-of-phase ESEEM experiments.

The simulations of the TrEPR spectra of *Gg*Cry4a WT and all three mutants are consistent with the formation of a FAD^•–^–TrpH^•+^ SCRP upon blue-light photoexcitation (Figure S8). While the simulation parameters for the three proteins with an unperturbed tryptophan tetrad are identical (Table S2), the W369F (W_D_F) mutant returns a significantly larger dipolar (through-space) coupling, *D*, between the FAD^•–^ and TrpH^•+^ radicals. The *r* ^–3^ dependence of *D* on the inter-radical separation, *r,* (in addition to the simulation parameters in Table S2) suggests a shortening of the electron transfer chain in W369F (*r* ≈ 17 Å) compared to the WT (*r* ≈ 21 Å) and R317C and E320K mutants. The difference in the fine structure of the TrEPR spectra of *Gg*Cry4a WT and W369F is, therefore, mainly attributed to a broadening of the spectrum due to this increase in the inter-radical dipolar coupling (*D*), in addition to a change in the relative orientation of the radicals, based on our simulations (see Figure S8 and Table S2). A truncated electron transfer cascade in *Gg*Cry4a W369F mirrors the TrEPR results in *Dm*Cry W394F, *Er*Cry4a W369F, and *Cl*Cry4a W369F.^18,24,26^

Full clarification of the identity of the terminal electron donor is provided by the gold standard technique for the determination of inter-radical distances in SCRPs, out-of-phase ESEEM. Figure 2c shows the out-of-phase ESEEM data obtained for *Gg*Cry4a WT, R317C, and E320K (purple, red, and green traces, respectively). The traces are, within signal-to-noise, identical, exhibiting clear modulations whose frequency is determined by the interactions between the two electron spins (SM.3). Full details of the out-of-phase ESEEM simulations in Figure 2c and 2d is provided in the Supporting Information (SM.3 and Figure S9), but Table 1 summarises the crucial parameters extracted here: the strengths of the dipolar coupling, *D*, and the resulting radical separations related by *D* (MHz) = −7.8 × 10^4^/[*r* (Å)]^3^. For all three proteins containing the full tryptophan tetrad, we obtain radical–radical separations of 21 ± 0.1 Å.

**Table 1:** Dipolar (*D*) Coupling Constants Obtained from Least-Squares Fitting to the Out-of-Phase ESEEM Traces Shown in Figure 2c and 2d^a^

Protein	*D*/MHz	*r*/Å
*Gg*Cry4a WT	–8.37 ± 0.06	21.04 ± 0.05
*Gg*Cry4a R317C	–8.46 ± 0.07	20.97 ± 0.06
*Gg*Cry4a E320K	–8.51 ± 0.07	20.93 ± 0.06
*Gg*Cry4a W369F	–13.95 ± 0.14	17.75 ± 0.06

a The inter-radical separations were calculated assuming the validity of the point-dipole approximation (see SM.3). The exponential damping, quantified by relaxation time, *T*_r_, and the exchange couplings, *J*, are provided in Table S4. .

For comparison, center-to-center separations between FAD and each W residue in the tetrad, as well as a neighboring tyrosine (Y), were extracted from a homology model of *Gg*Cry4a WT, based on the crystal structure of *Cl*Cry4a (Table S3).^34^ It is clear that electron transfer in *Gg*Cry4a WT, R317C, and E320K, occurs along the entire tryptophan tetrad, as in *Dm*Cry WT, *Cl*Cry4a WT and *Er*Cry4a WT, and in contrast to the triad of tryptophan residues in *At*Cry1 and *Ec*PL.^18,24–26,36^

Finally, Figure 2d shows the out-of-phase ESEEM trace for *Gg*Cry4a W369F. The higher modulation frequency is the result of a larger dipolar coupling from the shorter radical separation (18 Å) consistent with a radical pair containing FAD and W318 (W_C_, see Table S3).

### Transient Absorption Spectroscopy

Having established the identity of the RP1 radicals (Figure 1) in all four proteins, transient absorption (TA) spectroscopy was employed to trace the kinetics of various species produced by monitoring Δ*A* (= *A*_*hν*_ – *A*_GS_) as a function of the pump–probe delay time (see SM.4). *A*_*hν*_ and *A*_GS_ are the absorbance of the sample in the presence and absence (ground state absorbance) of a 450 nm photoexcitation beam, respectively.

The four panels in Figure 3 depict the time evolution of the wavelength-resolved Δ*A* spectra of the four proteins following laser flash illumination at time *t* = 0. Three dominating features are common to the spectral profiles of all four proteins (see also Figure S10a): (1) The negative ground state bleach (GSB) between 400 and 500 nm, matching the absorption profile of FAD_OX_ (with vibronic fine-structure indicating protein-bound FAD); (2) Positive Δ*A* signals indicative of the formation (and subsequent decay) of TrpH^•+^ and FAD^•–^ radicals (RP1), identified by their characteristic absorbance bands below 400 nm (FAD^•–^) and above 500 nm (initially mostly TrpH^•+^, with a minor contribution from FAD^•–^), respectively (cf. Figure S1). These radicals form on a femtosecond time scale beyond the time resolution of the spectrometer.^47^ (3) The subsequent decay of absorption signals on a submicrosecond time scale along with recovery in the GSB region are consistent with the recombination of radical pairs.

**Figure 3:**
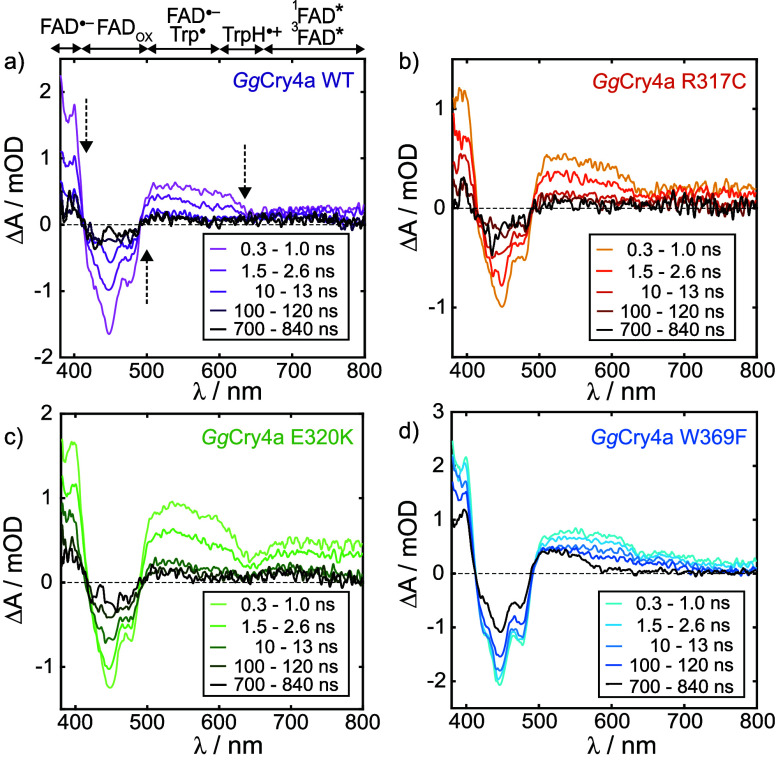
Wavelength-resolved transient absorption spectra, Δ*A*(λ), at different times following the 450 nm laser pulse at *t* = 0, for the air-equilibrated *Gg*Cry4a WT (a), *Gg*Cry4a R317C (b), *Gg*Cry4a E320K (c), and *Gg*Cry4a W369F (d) proteins. All spectra were averaged over the time-windows given in the corresponding legend. Above panel (a), a rough guide is provided indicating which species in the photoreaction scheme (Figure 1) contribute most to the spectra at a given wavelength. The feature between 500 and 650 nm is ascribed to absorption by FAD^•–^ and TrpH^•+^ (see Figure S1). The signals at wavelengths beyond 650 nm are assigned to singlet (^1^FAD*, 750–800 nm) and triplet (^3^FAD*, 650–720 nm) excited states of FAD with the former decaying more rapidly than the latter. We believe these signals derive from a small fraction of misfolded protein in which electron transfer along the Trp-tetrad is significantly slower than in the native state, allowing ^1^FAD* and, after intersystem crossing, ^3^FAD* to be detected. Similar signals were observed for European robin Cry4a by Xu et al. (ref 18, Figure S5). The assignment of the 650–720 nm signal to ^3^FAD*, rather than a radical intermediate, is supported by the absence of a magnetic field effect between 700 and 800 nm in GgCry4a W369F (see Figure S11).

The time evolution of the *Gg*Cry4a W369F spectrum (Figure 3d) is markedly different from the others and shows a longer-lived (*t* > 100 ns) component in the 500–600 nm region, which is a clear indication of Trp^•^ formation by deprotonation of the TrpH^•+^ radical. This results in the spin-uncorrelated radical pair, RP2 (see Figure 1).^18,20,37^ This feature is not observed in the TA spectra of the three proteins that have an intact electron transfer chain. The comparatively slow ground state recovery and radical absorbance decay above 500 nm in *Gg*Cry4a W369F, is thus a direct consequence of its efficient RP2 formation which, due to TrpH^•+^ deprotonation, cannot recombine directly to the ground state.

The temporal evolution of the Δ*A* signals for all four proteins, averaged over 500–550 nm, is shown in Figure 4a (and, with a logarithmic time-axis, in Figure S10b). The kinetic profiles in this wavelength region predominantly reflect the decay pathways of FAD^•–^ and Trp^•^/TrpH^•+^, and can be reliably fitted with a biexponential model for all four proteins plus a constant offset to account for the formation of long-lived photoproducts (Table 2).

**Figure 4:**
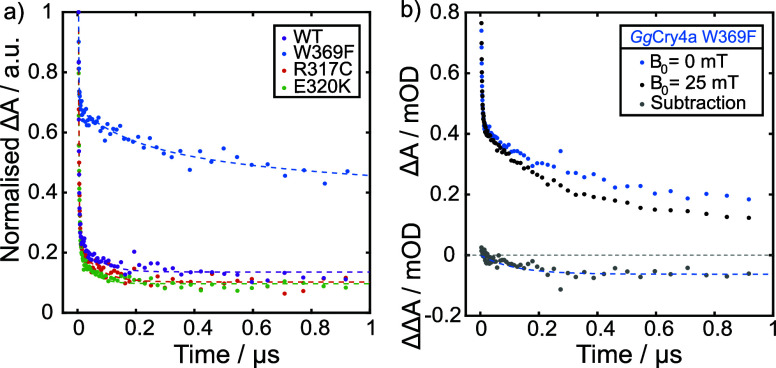
a) Normalized Δ*A*(*t*, *B*_0_ = 0) following the 450 nm laser pulse at *t* = 0, for air-equilibrated *Gg*Cry4a WT (purple), *Gg*Cry4a W369F (blue), *Gg*Cry4a R317C (red), and *Gg*Cry4a E320K (green) proteins, wavelength-averaged over 500–550 nm. The Δ*A* in this region is dominated by the absorbance of FAD^•‑^, TrpH^•+^, and Trp^•^. The dashed lines are kinetic fits to the data set of the same color. Fitting parameters are provided in Table 2. b) Δ*A*(t) traces of air-equilibrated *Gg*Cry4a W369F, averaged over 500–650 nm, obtained in the absence (blue) and presence (black) of a 25 mT external magnetic field. The ΔΔ*A*(*t*, *B*_0_) obtained from these data using eq 1 is shown in gray, below the Δ*A*(*t*) data. A monoexponential fit to the data including a constant offset (blue dashed line) yields a rise-time of 116 ± 16 ns for the development of the MFE. Further experimental details can be found in SM.4.

**Table 2:** Lifetimes Obtained for All Four Proteins from Kinetic Fitting of the Δ*A* Decay Curves, Averaged over 500–550 nm, Shown in Figure 4a^a^

Protein	τ_1_/ns	τ_2_/ns
*Gg*Cry4a WT	1.5 ± 0.5	60 ± 4.9
*Gg*Cry4a R317C	2.0 ± 0.5	64 ± 5.2
*Gg*Cry4a E320K	1.9 ± 0.5	40 ± 2.4
*Gg*Cry4a W369F	1.7 ± 0.5	299 ± 15

a All kinetic decays were fitted to bi-exponentials, including a constant offset to account for long-lived photoproducts. The uncertainty in τ_1_ is dominated by the pump pulse duration (1 ns) and that of τ_2_ is calculated from the error in the fit (see SM.3 for further details).

The faster (∼2 ns) components (τ_1_) should be interpreted with some caution as they approach the time resolution of the spectrometer. Nonetheless, they are similar in all four proteins and are assigned to the recombination of FAD^•–^ and the TrpH^•+^ at site 372 (W_B_). This is plausible as the efficiency of the electron transfer steps forming the terminal radical pair in *Er*Cry4a WT has recently been shown to decrease as charge separation proceeds along the tryptophan tetrad.^47^ The equivalent decay in the *Er*Cry4a W318F (W_C_F) mutant, also exhibited a dominant lifetime of 2.5 ns.^18^

The longer kinetic decay component (τ_2_) can be assigned to FAD^•–^ and TrpH^•+^ recombination involving the terminal tryptophan in each protein. As expected from the data in Figure 3d, τ_2_ is significantly larger in *Gg*Cry4a W369F compared to the other three proteins. The higher yield of species living beyond 1 μs reflects the efficient formation of RP2 in the *Gg*Cry4a W369F mutant.

The time constants for the decay of FAD^•–^ and TrpH^•+^ as well as the FAD_OX_ recovery in *Gg*Cry4 W369F may be estimated from the 370–400 nm, 600–650 nm, and 450–480 nm spectral regions, respectively (Figure S12a–c). This allows an estimate of the FAD^•–^ lifetime (τ_2, FAD^•–^_), the recovery time of the GSB (τ_2, FAD_OX__), and lifetime of W318 (W_C_) deprotonation (τ_2, TrpH^•+^_). All kinetic traces can be fitted to a biexponential decay plus a constant offset (Table S5). The short (∼2 ns) and long (275 ns, 193 ns, 270 ns, respectively) time constants agree well with those in Table 2. As both recombination and deprotonation time scales agree reasonably with the rate of GSB recovery, it appears that deprotonation of TrpH^•+^ and recombination of RP1 occur on similar time scales, and we assign the 299 ns component in Table 2 to a combination of these processes. These assignments are corroborated by the individual species’ kinetics of *Gg*Cry4 W369F (Figure S13) which clearly show the formation of Trp^•^ from TrpH^•+^ in conjunction with a partial decay in FAD^•–^ and GSB recovery, all on a time scale of ca. 200 ns (τ_2_ in Table S6).

Transient absorption spectroscopy has also been applied to study the field sensitivity of the radical pair dynamics. TA spectra were recorded in both the absence (Δ*A*(*t*, *B*_0_ = 0)) and presence (Δ*A*(*t*, *B*_0_)) of a static magnetic field, with the field effect quantified as,

$$\Delta \Delta A\left(t\right)=\Delta A\left(t,B_{0}\right)-\Delta A\left(t,B_{0}=0\right)$$ 1

As the proteins used in this work form singlet-born radical pairs (Figure 1) the application of fields greater than ∼1 mT (the effective hyperfine coupling) impedes singlet–triplet mixing, leading to an enhanced recombination yield and yield and lower radical concentrations.^48–50^ ΔΔ*A*(*t*) is, therefore, negative for a singlet-born radical pair.^19,20,35^

Figure 4b shows the time-resolved Δ*A*(*t*, *B*_0_ = 25 mT) (blue) and Δ*A*(*t*, 0 mT) (black) traces for *Gg*Cry4a W369F, averaged over 500–650 nm as well as their difference (ΔΔ*A*(*t*), gray). Three features of these temporal profiles are noteworthy. First, the application of a 25 mT field indeed leads to a significant decrease in the radical absorption over time. Second, this suppression in radical concentration develops after the sharp initial drop (τ_1_) in radical absorbance, and on a time scale comparable to τ_2_ (τ_ΔΔ*A*_= 116 ± 16 ns). This is not surprising as the fields applied here (25 mT, corresponding to 700 MHz electron Larmor frequency) have insufficient time to affect spin-mixing processes on the shorter (τ_1_ ∼ 2 ns) time scale. Third, the field effect plateaus after *ca*. 400 ns indicating formation of a photoproduct downstream of RP1 with a lifetime exceeding 1 μs (RP2).

No field effects were observed by TA in the proteins with intact tryptophan tetrads. This is explained by the fact that the majority (>80%) of the radicals produced in these proteins decay within 5 ns, and the recombination of the terminal tryptophan (W_D_) radical is also fast (40 ns). To characterize the field effects, we have utilized other optical techniques with superior sensitivity.

### Broadband Cavity-Enhanced Absorption Spectroscopy

We have developed broadband cavity-enhanced absorption spectroscopy (BBCEAS) to measure spectrally resolved magnetic field effects as low as 1% (ΔΔ*A* ≈ 1 μOD).^21,51^ A sample contained within an optical cavity is continually excited at 455 nm and changes in the absorbance monitored using a pseudocontinuous broadband supercontinuum probe beam. Field effects are measured with on/off magnetic fields. BBCEAS boasts a much-improved sensitivity over TA for accumulation of data on longer-lived photoproducts. As in TA spectroscopy, the Δ*A* and ΔΔ*A* signals quantify the effects of photoexcitation and applied magnetic fields on the cryptochrome samples, respectively.^18,21^

Figure 5a shows the evolution of the Δ*A* spectrum for *Gg*Cry4a WT, under constant illumination conditions. The GSB and radical absorbance both increase over the first 10 s of illumination as the photostationary equilibrium of the system is driven toward the photoproduct(s). Global spectral fitting of the kinetics (see Figure 5a inset and SM.8) confirm that the dominant absorbers on this time scale in the 500–750 nm region are the FADH^•^ and Trp^•^ radicals that comprise RP2. The species-resolved accumulation kinetics in Figure 5b shed light on the subsequent fate of the radicals: FAD^•–^ protonation on a ∼1 s time scale leads to steady accumulation of FADH^•^ over the illumination period, while the Trp^•^ concentration rises quickly before reaching a steady-state for *t* > 1 s. Similar behavior is observed in all four proteins studied (see Figure S14).

**Figure 5:**
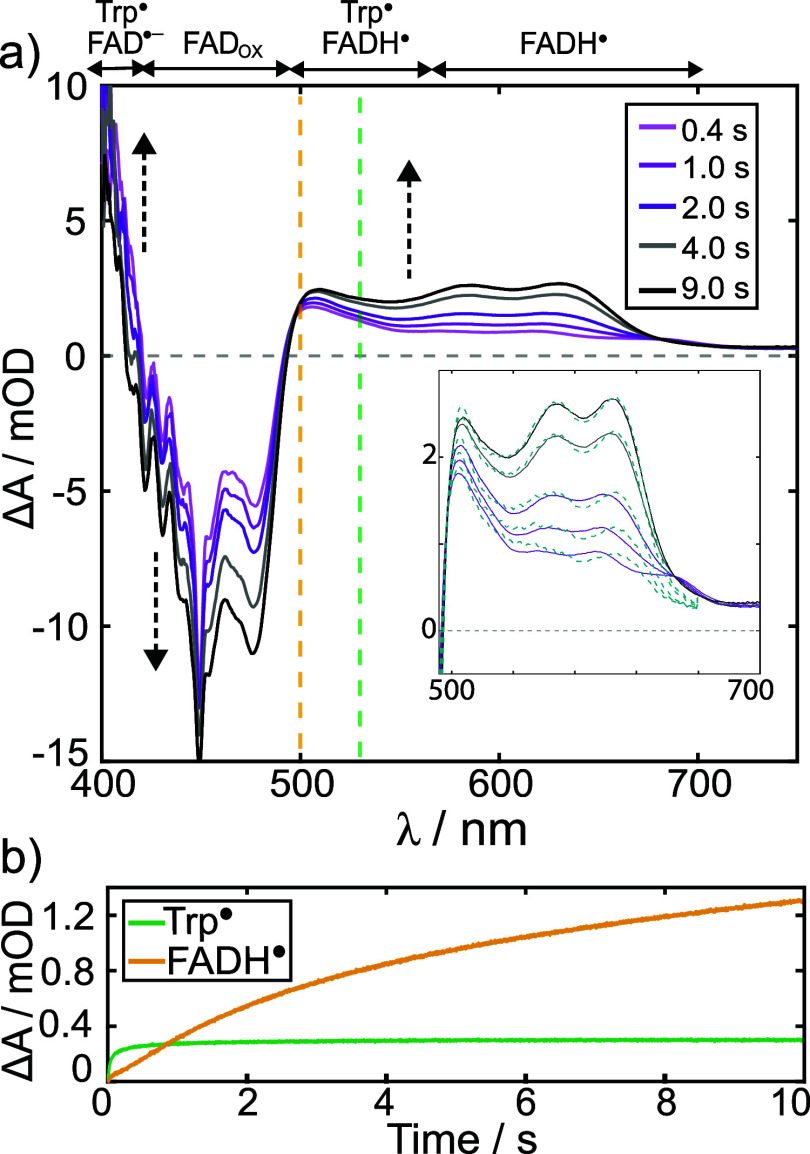
a) Δ*A* spectra obtained by BBCEAS for *Gg*Cry4a WT at the different time windows indicated in the legend. The yellow and light green dashed lines give the wavelengths at which the kinetics profiles are extracted in b). Dashed black arrows indicate the time evolution of the spectra. As in Figure 3a, a rough guide is provided indicating which species in the photoreaction scheme (Figure 1) contribute most to the spectra at a given wavelength. Inset: Spectral fit (teal dashed line) obtained by global analysis (see SM.8) of the BBCEAS Δ*A* spectra for *Gg*Cry4a WT (same as Figure 5a), using the literature spectra of FAD_OX_, FAD^•–^, FADH^•^, and Trp^•^ as basis spectra. b) Δ*A* accumulation kinetics of FADH^•^ at 500 nm (yellow) and Trp^•^ at 530 nm (light green) extracted by global fitting of the Δ*A* spectra (see inset and dashed lines) over the duration of the illumination period.

Figure 6a shows the wavelength-resolved, time-averaged ΔΔ*A* spectra (*B*_0_ = 30 mT) of all four proteins as recorded by BBCEAS. All exhibit negative MFEs across the 500–750 nm wavelength region confirming the formation of singlet-born SCRPs in each case. Under the O_2_-reoxidising conditions employed here, all ΔΔ*A* spectra were dominated by FADH^•^ with a smaller Trp^•^ contribution (see Figure S15).^51–53^ On this longer time scale, the 30 mT magnetic field has the largest effect on the concentration of the neutral flavin radical, FADH^•^, formed by protonation of FAD^•–^ following electron transfer.

**Figure 6:**
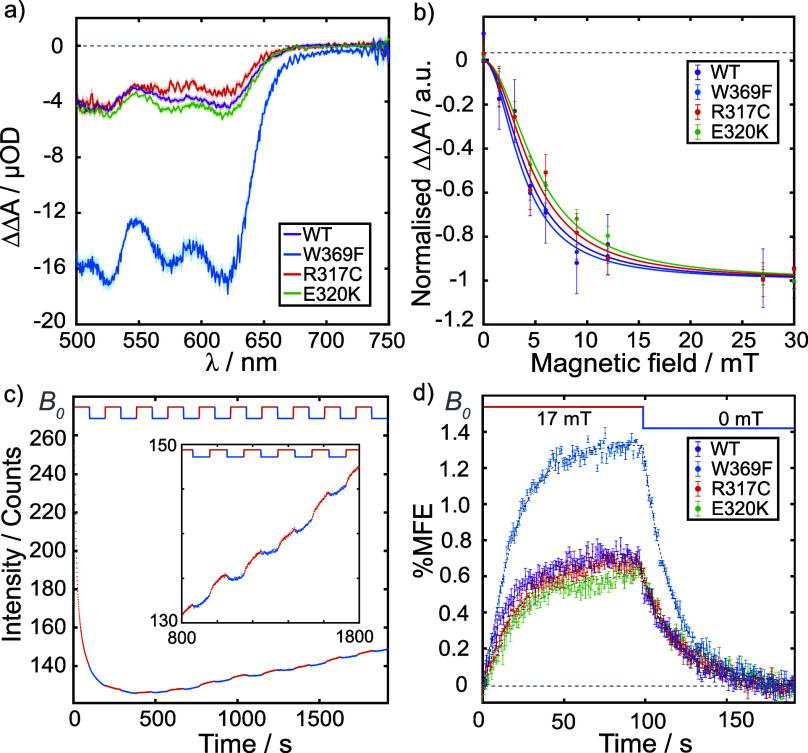
a) ΔΔ*A* spectra obtained by BBCEAS on all four proteins, averaged over 10 s, using an applied magnetic field strength of 30 mT and a field switching period of 200 ms. b) Magnetically altered reaction yield curves (solid circles), obtained by BBCEAS as in a), with varied applied magnetic field strength. The data were fit to a single Lorentzian (solid lines). c) Time evolution of the fluorescence intensity of *Gg*Cry4a WT, obtained by CM, averaged over the entire field of view (100 × 100 μm^2^), following onset of illumination (448 nm) at *t* = 0. A magnetic field with a period of 191.8 s was switched between 0 and 17 mT during the acquisition leading to modulations (see inset) in the fluorescence intensity during the field-on (red fluorescence segments) and field-off (blue fluorescence segments) periods. The applied magnetic field (*B*_0_) is shown schematically above the data, again with field-on and field-off periods denoted by red and blue. d) Time-averaged %MFE for all four proteins, obtained by CM, calculated using eq 2, from background subtracted data (Figure S16). The black dashed lines are single-exponential fits to the mean %MFE over both the field-on and field-off periods. The time constants (τ_eMFE_) reported in Table 3 were obtained from these fits.

The *Gg*Cry4a WT, R317C, and E320K proteins all exhibit very similar MFEs (ΔΔ*A*_500–630 nm_ ≈ −4 μOD). The field effect on the W369F mutant, is substantially larger (ΔΔ*A*_500–630 nm_ ≈ −16 μOD), consistent with the differences between *Er*Cry4a WT and *Er*Cry4a W369F reported previously.^18,35^

Figure 6b shows the wavelength-averaged (500–630 nm) ΔΔ*A* signal for all four proteins as a function of the strength of the applied static magnetic field. All four species exhibit a Lorentzian-like dependence on *B*_0_, characteristic of hyperfine coupling-driven singlet–triplet mixing in a SCRP. These magnetically altered reaction yield (MARY) curves are commonly characterized by their half width at half-maximum, a parameter known as *B*_1/2_. Broadly, this parameter reflects the extent of hyperfine coupling within the SCRP via the Weller formula, which, for an FAD-Trp SCRP, predicts a *B*_1/2_ of approximately 3 mT.^54^ Wong et al. have estimated an improved lower bound for *B*_1/2_ in cryptochromes under our *in vitro* conditions of 2.46 mT.^50^ All four species exhibit similar values of *B*_1/2_ (≈ 4 mT, Table S6). This is consistent with an FAD^•–^/TrpH^•+^ SCRP with some spin relaxation impacting the dynamics during the radical pair lifetime, leading to a larger *B*_1/2_ than the idealized values mentioned above.^54,55^ In summary, the sensitivity of BBCEAS helps confirm the formation of magnetically sensitive radical pairs in all four proteins with very similar *B*_1/2_ values. Furthermore, mutations near the tryptophan chain have little detectable effect on the size of the MFE and removal of W_D_ leads to a significant increase in the size of the field effect, mirroring earlier results for *Er*Cry4a.^18^

### Confocal Microscopy

While EPR and the direct absorption techniques above probe the photoproducts (right-hand side of the photoreaction scheme in Figure 1a), Confocal Microscopy (CM) yields complementary information on the fluorescence of the flavin excited state. Photoexcitation of FAD is followed by rapid (< ns) intramolecular electron transfer along the tryptophan chain resulting in a very small fluorescence quantum yield.^56,57^ However, pseudocontinuous photoexcitation of the sample accumulates ground state FAD_OX_ that has undergone repeated RP singlet–triplet mixing cycles. Any magnetic field effect on RP1 directly impacts the FAD fluorescence intensity.^58–60^ Combined with sensitive fluorescence detection, this makes CM a powerful method for studying MFEs on cryptochromes.^60,61^

Following the approach described in ref 60, protein samples were subject to continuous 448 nm (raster-scanned) laser excitation (see SM.6). An external magnetic field was switched on and off repeatedly during the acquisition to probe the MFE on the sample fluorescence. Importantly, increased RP1 recombination arising from an applied magnetic field *increases* the concentration of ground state FAD_OX,_ and, hence, the observed fluorescence. This approach to field-effect detection of a flavoprotein was first demonstrated with *At*Cry1.^58–60^ Figure 6c depicts the CM-detected fluorescence intensity of *Gg*Cry4a WT, averaged over the entire field of view, as a function of time following the onset of 448 nm photoillumination at *t* = 0. The fluorescence intensity exhibits an initial sharp drop ascribed to photobleaching of the FAD_OX_ cofactor. It then slowly increases again as a result of two processes: photoinduced degradation and diffusion of fresh protein into the field-of-view, with the former process dominating at long times.^58,60^ The former process results in the release of unbound FAD, which, lacking a nearby electron donor, exhibits a much increased fluorescence quantum yield (ca. ten times higher relative to protein-bound FAD).^56,62^ Superimposed on the time evolution described above is a shallow modulation of the fluorescence intensity, in synchrony with the switching of an applied static field (Figure 6c, top) between 17 mT (red) and 0 mT (blue).

The background fluorescence was subtracted (Figure S16a-b) to obtain a %MFE, defined as

$$\%\mathrm{MFE}=\frac{I_{F}\left(B_{0}\right)-I_{F}\left(B_{0}=0\right)}{I_{F}\left(B_{0}=0\right)}\times 100$$ 2

where *I*_F_(*B*_0_) and *I*_F_(*B*_0_ = 0) are the fluorescence intensities in the presence and absence of a magnetic field, respectively.

Close inspection of Figure 6c reveals a consistent increase of the fluorescence intensity with an applied magnetic field. Averaging of several “field on–off” cycles (Figure S16c) gives insight into the dynamics of the field effect evolution. Figure 6d shows the time-averaged %MFE data for all four proteins. As expected for singlet-born radical pairs, the applied magnetic field hinders singlet–triplet mixing leading to more singlet recombination to the ground state and an increase in fluorescence. The %MFE of all four Cry4a proteins displays a delayed response to the application of a magnetic field. This is characteristic of enhanced magnetic field effects (eMFEs), leading to biphasic kinetics of the MFE; a prompt fluorescence (increase) upon magnetic field application followed by a slow rise to a constant value. eMFEs were first studied in the solution phase in ref 58 but have since been reported in both fluorophore-doped protein crystals^60^ and human cells^63^ (note that the failure of ref 64 to reproduce the latter has been rebutted by the authors of the original study in ref 65). This is, however, the first example of eMFEs in an avian cryptochrome. These eMFEs are similar to those previously reported for *At*Cry1 and are consistent with *k*_F_ > *k*_W_ (see Figure 1).^58^

The eMFEs of all four proteins can be fit well by a single exponential function with a time constant, τ_eMFE_ (Table 3), which is the same for the field-on and field-off steps. All four eMFEs saturate by ca. 50 s after magnetic field switching, with a reproducibly faster τ_eMFE_ for *Gg*Cry4a W369F relative to the other three proteins, which are very similar to one another.

**Table 3:** Time Constants Obtained by Fitting an Exponential to the Delayed Component of the %MFE Determined by CM for All Four Proteins

Protein	τ_eMFE_/s
*Gg*Cry4a WT	21.0 ± 0.4
*Gg*Cry4a R317C	23.3 ± 0.3
*Gg*Cry4a E320K	23.3 ± 0.5
*Gg*Cry4a W369F	17.7 ± 0.2

The three proteins containing a complete tryptophan tetrad exhibit the same %MFEs within signal-to-noise. *Gg*Cry4a W369F (Figure 6d, blue), by contrast exhibits a significantly more pronounced field sensitivity, paralleling the results from BBCEAS and TA. The trends in the relative magnitudes of the MFEs of all four samples studied in this work are, therefore, reproducible between all spectroscopic techniques employed here, probing complementary parts of the photocycle.

### Continuous Blue-Light Illumination

All the methods described thus far rely on the recovery of the system back to the ground state before re-excitation of the flavin leading to formation of the magnetosensitive radical pair, RP1. To test the samples for their behavior under long-term illumination, we exposed the proteins to 1 min of light of 450 nm (ca. 25 W m^–2^) and followed their recovery using UV–visible spectroscopy. Figure 7a (purple line) shows the UV–visible spectrum of *Gg*Cry4a WT after 1 min illumination. The pronounced 500–700 nm absorption reflects the generation of the stable protonated flavin semiquinone (FADH^•^).^18,26^

**Figure 7:**
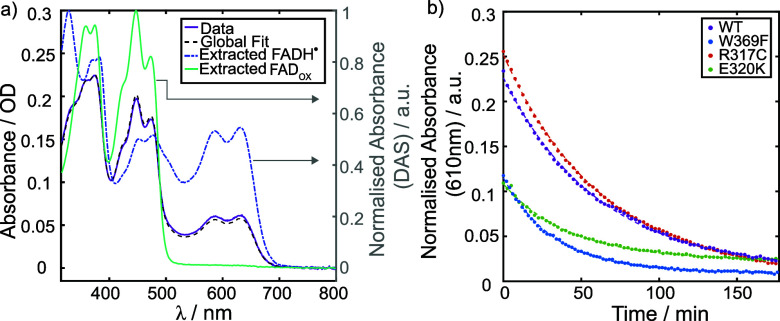
a) UV–visible spectrum of *Gg*Cry4a WT (purple) after 1 min illumination at 450 nm. The spectrum was successfully modeled by global analysis (black dashed line) assuming a single first-order recovery (see SM. 8). The normalized decay associated spectra (DAS) obtained from kinetic fitting are consistent with recovery from FADH^•^ (blue dashed spectrum) to FAD_OX_ (teal spectrum). b) Recovery kinetics of FADH^•^, averaged over 605–615 nm, for *Gg*Cry4a WT (purple), *Gg*Cry4a W369F (blue), *Gg*Cry4a R317C (red), and *Gg*Cry4a E320K (green), with photoexcitation ceasing at *t* = 0. The kinetics are normalized to the absorbance maximum of FAD_OX_ at 450 nm, prior to sample illumination. Single exponential fits to each kinetic trace are given by dashed lines of the same color as the data.

This assignment is confirmed by a global analysis fit (black dashed line, see SM.8) obtained assuming a single first-order recovery from FADH^•^ (dashed blue spectrum) to FAD_OX_ (solid teal spectrum). The blue and teal spectra are decay associated spectra (DAS) obtained by the global analysis procedure. As with other avian cryptochromes, these systems are efficiently photoreduced under moderate light intensity.^18,26^

Figure 7b shows the recovery kinetics of FADH^•^, obtained at 298 K (25 °C) by averaging over the 605–615 nm region. The relative FADH^•^ radical yield is larger in *Gg*Cry4a WT (purple) and R317C (red) than in W369F (blue) or E320K (green). This suggests that the replacement of W_D_ by phenylalanine and the presence of K320 affects the efficiency of formation and/or lifetime (see Table 4) of the semiquinone radical. This observation is consistent with other measurements on single-point mutants of *Dm*Cry, producing long-lived FAD^•–^, due to differences in the redox states stabilized by these proteins.^37,66^ In the *Dm*Cry measurements, mutations to the tryptophan chain as well as residues neighboring the terminal tryptophan decreased both the rate of photoreduction under steady-state conditions and the overall photoreduction yield.^25,37,66^

**Table 4:** Time Constants (*τ*_rec_) Obtained by Monoexponential Fitting of the Decay of FADH^•^ Generated by Continuous Blue-Light Illumination, Shown in Figure 7b

Protein	τ_rec_/min
*Gg*Cry4a WT	61.4 ± 0.21
*Gg*Cry4a R317C	63.9 ± 0.15
*Gg*Cry4a E320K	42.8 ± 0.32
*Gg*Cry4a W369F	32.6 ± 0.15

The lifetimes obtained by kinetic fitting of the FADH^•^ recovery data to a single exponential including constant offsets (Table 4, and Figure 7b) agree well with those obtained by global analysis (Table S7). The fastest recoveries were observed for *Gg*Cry4a W369F and *Gg*Cry4a E320K (τ_rec_ = 33 min and τ_rec_ = 43 min, respectively). The time scale on which these proteins recover is in general agreement with those obtained in ref 17, with differences due to the choice of buffer and illumination conditions (see SM.7).

## Discussion

The focus of this study was a comparative, spectroscopic investigation of four Cry4a avian cryptochromes, namely wild-type *Gg*Cry4a and three of its mutants, each with a single-point mutation. These were W369F, in which W_D_ is replaced by a phenylalanine, and R317C and E320K, both chosen after sequence comparison had highlighted amino acid positions 317 and 320 as distinct between migratory (C317 and K320) and nonmigratory birds, such as the chicken (R317 and E320).^11^ The most notable observations of our comprehensive study are:

### 1 All Four Proteins Form Spin-Correlated Radical Pairs

The TrEPR data of all four proteins (Figure 2) show the typical (fingerprint) antiphase structure characteristic for SCRPs. Furthermore, these spectra are consistent with an FAD^•–^ TrpH^•+^ radical pair (RP1), with differences between the spectra of *Gg*Cry4a WT/R317C/E320K relative to *Gg*Cry4a W369F attributed to different relative orientations of the FAD^•–^ and TrpH^•+^ radicals. The inter-radical separation between FAD^•–^ and the terminal TrpH^•+^ is markedly shorter in *Gg*Cry4a W369F than the other proteins, as confirmed by out-of-phase ESEEM measurements.^18,24^ This demonstrates that electron transfer in wild-type *Gg*Cry4a proceeds along the whole tetrad of tryptophans as in *Dm*Cry, *Cl*Cry4a, and *Er*Cry4a.^18,20,24–26^ EPR also confirms that the inter-radical separation and coupling parameters (|*D*| = 8–13 MHz, *J* ≈ 0 MHz) are sufficiently small in all four proteins to allow efficient singlet–triplet interconversion at the field strengths employed here leading to magnetic field effects on the radical recombination yields and kinetics.

### 2 Shortening of the Electron Transfer Chain Increases the Lifetime of the Radical Pair, RP1

TA spectroscopy (Figure 3 and Table 2, τ_2_) indicates that the radical pair lifetime of RP1 in the (W369F) mutant protein with its triad of tryptophan residues exceeds that of the proteins with the full tetrad, *in vitro*. This result seems counterintuitive, given the shorter inter-radical distance of the terminal FAD^•–^ TrpH^•+^ radical pair which leads to faster radical recombination. One explanation for this unexpected result is that the back electron transfer, which returns RP1 to the ground state, is in the Marcus inverted region.^67^ Thus in *Gg*Cry4a W369F the radical recombination is slowed by an increase in the driving force for back-electron transfer or a significant decrease in the reorganization energy (due e.g. to a change in the solvation of the tryptophan radical). Few studies of electron transfer in cryptochromes have been reported, but detailed ultrafast investigations of related flavoproteins (including *Ec*PL and *At*Cry3) have found that all back electron transfer processes involving the tryptophan triad are in the Marcus inverted region.^68,69^ This work supports an analogous process in avian Cry4a.

### 3 Shortening of the Electron Transfer Chain Increases the Effect of Applied Magnetic Fields on the Radical Kinetics and Yields

In agreement with previous results on *Er*Cry4a W369F, shortening of the tryptophan chain to three residues in GgCry4a W369F increases the protein’s magnetic field sensitivity *in vitro*, as measured by all the spectroscopy methods employed here.^18^ As observed previously for *Ec*PL, this results from similar time scales of protonation and terminal tryptophan recombination (Figure 4a, Figure S12) in *Gg*Cry4a W369F, resulting in larger MFEs than the other three proteins (Figure 4b, Figure 6a,d).^20^ The similar deprotonation time scales in *Gg*Cry4a W369F and *Dm*Cry W394F mutants (both lacking their W_D_, with W318 (W_C_) acting as the terminal donor) supports the kinetic assignments of the TA spectra (Figure S12, Table S5).^24^ The rate of deprotonation is, however, an order of magnitude shorter than the reported RP2 formation rate in *Ec*PL (with its tryptophan triad).^20,25,44^ However, the study on *Ec*PL used higher glycerol concentration than the work conducted here, which could, in part, account for the prolonged lifetime of TrpH^•+^. It may also indicate that TrpH^•+^ at residue 318 (W_C_) in avian Cry4a’s is more liable to deprotonation than the corresponding 306 (W_C_) position in *Ec*PL.

In contrast to *At*Cry1 and *Ec*PL^20^ no clear evidence was found here for Low Field Effects (in which the sign of the MFE is inverted when the magnetic field is weaker than the effective hyperfine interaction).^70^ The same is true for *Er*Cry4a.^18^ Compared to ref 20, the measurements reported here were performed under conditions closer to those expected *in vivo*, specifically higher temperature (278 K vs 250 K), which may have prevented the emergence of a sign change. It is possible that a strong Low Field Effect only occurs when the protein is aligned by complexation to membrane proteins in photoreceptor cells in the retina.^50^

### 4 Mutations of Amino Acid Positions 317 and 320 Do Not Impact Significantly on Radical Pair Kinetics or Magnetic Field Effects; Minor Changes Are Observed for FADH^•^ Reoxidation

All spectroscopies employed here confirm that mutation of neither R317 nor E320 to cysteine and lysine, respectively, has significant impact on either the radical pair kinetics or the magnetic field effects *in vitro*.

In continuous illumination experiments, minor differences in the FADH^•^ lifetime are observed for the E320K mutant compared to the WT and R317C (Figure 7b). This could be due to the proximity of the lysine to the W_D_ altering the reduction potential of Trp^•^, such that it is less easily reduced, thus promoting bimolecular recombination of FADH^•^ with Trp^•^. Support for such a mechanism comes from the fact that lysine residues modify the redox chemistry of iron–sulfur clusters in fumarate reductase.^71^ Further work on this hypothesis is presently ongoing but is outside the scope of this paper.

In W369F at 298 K (25 °C), FADH^•^ recovers more rapidly than in the WT following 1 min photoillumination (Figure 7b, Table 4). Additionally, a smaller τ_eMFE_ relative to the other three proteins is observed by CM. This is perhaps expected, given that the environment of the Trp^•^ is significantly perturbed in this mutant and that τ_eMFE_ is determined by the lifetimes of the flavin and tryptophan radicals. The τ_eMFE_ values obtained here are significantly longer than those reported for *At*Cry1 using a wide-field microscopy.^58^ We attribute this to raster scanning of the excitation laser employed in CM, slowing the MFE kinetics. It is noteworthy that a prolonged τ_eMFE_ is also observed for *At*Cry1 in CM (Figure S17).

The faster recovery of W369F in both CM and continuous illumination experiments can be explained by concomitant recovery of FADH^•^ and Trp^•^, with any solvent mediated recovery of Trp^•^ suppressed, as W318 (W_C_) is less solvent exposed than W369 (W_D_). The shorter τ_eMFE_ provides an additional explanation for the much larger MFE observed in *Gg*Cry4a W369F by BBCEAS. While the field switching time employed in BBCEAS is too fast to resolve eMFEs, the time evolution of the MFE over the 100 ms field-on time still contributes to the overall BBCEAS ΔΔ*A* signature. A faster τ_eMFE_ in this system leads to an amplification of the MFE, relative to the other three proteins.

### 5 Oxygen Efficiently Recycles Cry4a Proteins under Continuous or Repeated Light Exposure

Our results provide the first demonstration that photoreduced Cry4a’s can be effectively recycled by molecular oxygen *in vitro* (see SM.3–7 for details) and that MFEs can be detected without adding oxidizing agents, e.g. ferricyanide. The exact reoxidation mechanism is currently unknown. Studies of *At*Cry1 have suggested superoxide (O_2_^•–^) as a possible reaction partner for FADH^•^, generating hydrogen peroxide as a biproduct of flavin oxidation,^72^ consistent with similar models proposed for flavoenzymes, including monooxygenases.^73,74^ A test of this hypothesis could involve addition of peroxide scavengers to samples of Cry4a exposed to different O_2_ concentrations and/or varied duration and intensity of light exposure. Such measurements have been conducted on *Dm*Cry using *in vitro* fluorescence assays confirming the generation of reactive oxygen species upon blue-light illumination.^75^ However, it is unlikely that the superoxide radicals would contribute to the spin dynamics of the systems due to rapid spin-relaxation of any resulting radical pair, driven by spin–orbit coupling in O_2_^•–^.^76^ Kattnig and colleagues^77,78^ have proposed a mechanism that could circumvent this issue via a paramagnetic scavenger that could react spin-selectively with one of the components of a flavin-superoxide radical pair. Probably the best way to test this idea would be to attach a stable radical to the protein with appropriate redox properties at a position predicted by spin dynamics simulations.^77,78^

## Conclusion

Despite their evolutionary conservation, the cysteine and lysine residues in positions 317 and 320 in *Er*Cry4a have little impact on either the spin dynamics or radical pair kinetics when introduced into *Gg*Cry4a. Any differences in the magnetic sensitivity of robin and chicken Cry4a's (and, more generally, between Cry4a proteins from migratory and nonmigratory birds) must therefore lie in their magnetic *signaling*, rather than *sensing*, properties. Positions 317 and 320 are both solvent-exposed and so could facilitate the binding of Cry4a to intracellular signaling partners, some of which have been provisionally identified.^79,80^ More information regarding the entire signal transduction process will be required to reach definite conclusions on the role of these sequence differences. The lysine residue does affect recovery times of FADH^•^, and flavin redox states such as FADH^•^ and FADH^–^ are known to act as biologically relevant states in *At*Cry1/*At*Cry2 and *Ec*PL, respectively. This residue could be used to tune the flavin lifetime for this purpose.^81^ Other residues may be involved in light-induced structural rearrangements, e.g. in the C-terminal domain.^82^

Mutation of the fourth tryptophan to a phenylalanine (*Gg*Cry4a W369F) has a dramatic effect in all spectroscopic studies employed here. EPR demonstrates a shortening of the electron transport chain in W369F compared to the WT and confirms that RP1 is FAD^•–^ paired with the fourth (W_D_) tryptophan in the WT protein. Furthermore, all the techniques used here find an increase in the protein’s magnetic-field sensitivity for the W369F mutant. The same result was observed for *Er*Cry4a,^18^ and suggests that, while nature has seemingly optimized the avian magnetic compass to use four tryptophan residues, this is at the expense of the magnetic sensitivity, at least *in vitro*. This may point to a role for the solvent-exposed tryptophan residue, W_D_, in signaling, with suggestions that Cry4a may stabilize a composite radical pair, in which the magnetic sensitivity is optimized through the spin-dependent reactions of W318 (W_C_) and signaling involving W369 (W_D_). These possibilities have been discussed in detail in refs. 18 and 83. More detailed experimental investigations, including temperature-dependent out-of-phase ESEEM studies and single-point mutations (e.g., tryptophan to tyrosine) which could perturb the energetics of the equilibrium would be required to confirm such a hypothesis.

Finally, we believe our work represents an important step forward, providing a new gold standard for spectroscopic investigations of Cry4a proteins under more biologically relevant conditions, avoiding the harsh oxidizing conditions employed (such as the use of potassium ferricyanide).^18,21,66^

## Supplementary Material



## Notes

Experimental and Data Analysis Methods, protein characterization by native mass spectrometry and UV–vis spectroscopy, additional EPR, TA and BBCEAS spectra and CM data, global analysis fitting procedure and results for BBCEAS and TA spectroscopy, comparison of Cry4a sequences of migratory and nonmigratory birds, comparison of EPR and BBCEAS data acquired with and without potassium ferricyanide (PDF)

## References

[1] Kaptein R., Oosterhoff J. L. (1969). Chemically Induced Dynamic Nuclear Polarization II:
(Relation with Anomalous ESR Spectra). Chem.
Phys. Lett..

[2] Schulten K., Swenberg C. E., Weller A. (1978). A Biomagnetic Sensory Mechanism Based
on Magnetic Field Modulated Coherent Electron Spin Motion. Zeitschrift für Physikalische Chemie.

[3] Wiltschko W., Munro U., Ford H., Wiltschko R. (1993). Red Light
Disrupts Magnetic Orientation of Migratory Birds. Nature.

[4] Zapka M. (2009). Visual but not Trigeminal
Mediation of Magnetic Compass Information
in a Migratory Bird. Nature.

[5] Hein C. M., Zapka M., Heyers D., Kutzschbauch S., Schneider N.-L., Mouritsen H. (2010). Night-migratory
Garden Warblers Can
Orient with their Magnetic Compass Using the Left, the Right or Both
Eyes. J. R. Soc. Interface.

[6] Gunther A., Einwich A., Sjulstok E., Feederle R., Bolte P., Koch K.-W., Solov’yov I. A., Mouritsen H. (2018). Double-Cone
Localization and Seasonal Expression Pattern Suggest a Role in Magnetoreception
for European Robin Cryptochrome 4. Curr. Biol..

[7] Chetverikova R., Dautaj G., Schwigon L., Dedek K., Mouritsen H. (2022). Double Cones
in the Avian Retina Form an Oriented Mosaic which Might Facilitate
Magnetoreception and/or Polarized Light Sensing. J. R. Soc. Interface.

[8] Ahmad M., Cashmore A. R. (1993). HY4 Gene of *A. thaliana* Encodes a
Protein with Characteristics of a Blue-light Photoreceptor. Nature.

[9] Ozturk N. (2017). Phylogenetic
and Functional Classification of the Photolyase/Cryptochrome Family. Photochem. Photobiol..

[10] Lin C., Todo T. (2005). The Cryptochromes. Genome Biol..

[11] Langebrake C., Manthey G., Frederiksen A., Lugo Ramos J. S., Dutheil J. Y., Chetverikova R., Solov'yov I. A., Mouritsen H., Liedvogel M. (2024). Adaptive Evolution
and Loss of a
Putative Magnetoreceptor in Passerines. Proc.
R. Soc. B: Biol. Sci..

[12] Wiltschko, R. ; Wiltschko, W. Magnetic Orientation in Animals; Springer: Berlin, Heidelberg, 1995; Vol. 33.

[13] Mouritsen H. (2018). Long-distance
Navigation and Magnetoreception in Migratory Animals. Nature.

[14] Ritz T., Thalau P., Phillips J. B., Wiltschko R., Wiltschko W. (2004). Resonance Effects Indicate a Radical-Pair Mechanism
for Avian Magnetic Compass. Nature.

[15] Leberecht B., Wong S. Y., Satish B., Doge S., Hindman J., Venkatraman L., Apte S., Haase K., Musielak I., Dautaj G., Solov’yov I. A., Winklhofer M., Mouritsen H., Hore P. J. (2023). Upper Bound for Broadband Radiofrequency
Field Disruption of Magnetic Compass Orientation in Night-migratory
Songbirds. Proc. Natl. Acad. Sci. U. S. A..

[16] Engels S. (2014). Anthropogenic Electromagnetic
Noise Disrupts Magnetic Compass Orientation
in a Migratory Bird. Nature.

[17] Hore P. J., Mouritsen H. (2016). The Radical-pair
Mechanism of Magnetoreception. Annual Review
of Biophysics.

[18] Xu J. (2021). Magnetic Sensitivity of Cryptochrome 4 from a Migratory Songbird. Nature.

[19] Henbest K. B. (2008). Magnetic-field Effect on the Photoactivation
Reaction of *Escherichia coli* DNA photolyase. Proc.
Natl. Acad. Sci. U. S. A..

[20] Maeda K. (2012). Magnetically Sensitive Light-induced Reactions in Cryptochrome are
Consistent with its Proposed Role as a Magnetoreceptor. Proc. Natl. Acad. Sci. U. S. A..

[21] Sheppard D. M. W., Li J., Henbest K. B., Neil S. R. T., Maeda K., Storey J., Schleicher E., Biskup T., Rodriguez R., Weber S., Hore P. J., Timmel C. R., Mackenzie S. R. (2017). Millitesla
Magnetic Field Effects on the Photocycle of an Animal Cryptochrome. Sci. Rep..

[22] Evans E. W. (2013). Magnetic Field Effects
in Flavoproteins and Related Systems. Interface
Focus.

[23] Oldemeyer S., Mittag M., Kottke T. (2019). Time-Resolved
Infrared and Visible
Spectroscopy on Cryptochrome aCRY: Basis for Red Light Reception. Biophys. J..

[24] Nohr D. (2016). Extended Electron-transfer in Animal Cryptochromes
Mediated by a
Tetrad of Aromatic Amino Acids. Biophys. J..

[25] Nohr D. (2017). Determination of Radical–radical
Distances in Light-active
Proteins and Their Implication for Biological Magnetoreception. Angew. Chem., Int. Ed..

[26] Hochstoeger T., Al Said T., Maestre D., Walter F., Vilceanu A., Pedron M., Cushion T. D., Snider W., Nimpf S., Nordmann G. C., Landler L., Edelman N., Kruppa L., Durnberger G., Mechtler K., Schuechner S., Ogris E., Malkemper E. P., Weber S., Schleicher E., Keays D. A. (2020). The Biophysical, Molecular, and Anatomical Landscape
of Pigeon Cry4: A Candidate Light-based Quantal Magnetosensor. Sci. Adv..

[27] Hein C. M., Engels S., Kishkinev D., Mouritsen H. (2011). Robins Have
a Magnetic Compass in Both Eyes. Nature.

[28] Mora C. V., Davison M., Martin Wild J., Walker M. M. (2004). Magnetoreception
and its Trigeminal Mediation in the Homing Pigeon. Nature.

[29] Waterhouse A. (2018). SWISS-MODEL: Homology Modelling of Protein Structures and Complexes. Nucleic Acids Res..

[30] Guex N., Peitsch M. C., Schwede T. (2009). Automated Comparative Protein Structure
Modeling with SWISS-MODEL and Swiss-PdbViewer: A Historical Perspective. Electrophoresis.

[31] Studer G. (2020). QMEANDisCo—distance Constraints Applied on Model Quality Estimation. Bioinformatics.

[32] Bertoni M., Kiefer F., Biasini M., Bordoli L., Schwede T. (2017). Modeling Protein
Quaternary Structure of Homo- and Hetero-oligomers beyond Binary Interactions
by Homology. Sci. Rep..

[33] Bienert S. (2017). The SWISS-MODEL Repository—New Features and
Functionality. Nucleic Acids Res..

[34] Zoltowski B. D. (2019). Chemical and Structural
Analysis of a Photoactive Vertebrate Cryptochrome
from Pigeon. Proc. Natl. Acad. Sci. U. S. A..

[35] Golesworthy M. J., Zollitsch T., Luo J., Selby D., Jarocha L. E., Henbest K. B., Pare-Labrosse O., Bartolke R., Schmidt J., Xu J., Mouritsen H., Hore P. J., Timmel C. R., Mackenzie S. R. (2023). Singlet–triplet
Dephasing in Rradical Pairs in Avian Cryptochromes Leads to Time-dependent
Magnetic Field Effects. J. Chem. Phys..

[36] Giovani B., Byrdin M., Ahmad M., Brettel K. (2003). Light-induced Electron
Transfer in a Cryptochrome Blue-light Photoreceptor. Nat. Struct Mol. Biol..

[37] Paulus B. (2015). Spectroscopic Characterization of Radicals and Radical Pairs in Fruit
Fly Cryptochrome–Protonated and Nonprotonated Flavin Radical-states. FEBS Journal.

[38] Hore P. J., Hunter D. A., McKie C. D., Hoff A. J. (1987). Electron paramagnetic
resonance of spin-correlated radical pairs in photosynthetic reactions. Chem. Phys. Lett..

[39] Buckley C. D., Hunter D. A., Hore P. J., McLauchlan K. A. (1987). Electron
Spin Resonance of Spin-correlated Radical Pairs. Chem. Phys. Lett..

[40] Bittl R., Weber S. (2005). Transient Radical Pairs Studied by
Time-resolved EPR. Biochim. Biophys. Acta.

[41] Salikhov K. M., Kandrashkin Yu. E., Salikhov A. K. (1992). Peculiarities of Free Induction and
Primary Spin Echo Signals for Spin-correlated Radical Pairs. Appl. Magn. Reson..

[42] Biskup T. (2011). Unexpected Electron Transfer in Cryptochrome Identified by Time-resolved
EPR Spectroscopy. Angew. Chem., Int. Ed..

[43] Biskup T. (2009). Direct Observation of
a Photoinduced Radical Pair in a Cryptochrome
Blue-light Photoreceptor. Angew. Chem., Int.
Ed..

[44] Park H.-W., Kim S.-T., Sancar A., Deisenhofer J. (1995). Crystal Structure
of DNA Photolyase from Escherichia coli. Science.

[45] Biskup T. (2013). Variable Electron Transfer
Pathways in an Amphibian Cryptochrome. J. Biol.
Chem..

[46] Hore, P. J. Chapter 12 - Analysis of Polarized EPR Spectra. In Advanced EPR; Hoff, A. J. , Ed.; Elsevier: Amsterdam, 1989; pp 405–440. 10.1016/B978-0-444-88050-5.50017-3.

[47] Timmer D. (2023). Tracking the Electron Transfer Cascade in European Robin Cryptochrome
4 Mutants. J. Am. Chem. Soc..

[48] Timmel C. R., Henbest K. B. (2004). A Study of Spin
Chemistry in Weak Magnetic Fields. Philosophical
Transactions of the Royal Society of London.
Series A: Mathematical, Physical and Engineering Sciences.

[49] Steiner U. E., Ulrich T. (1989). Magnetic Field Effects
in Chemical Kinetics and Related
Phenomena. Chem. Rev..

[50] Wong S. Y., Benjamin P., Hore P. J. (2023). Magnetic
Field Effects on Radical
Pair Reactions: Estimation of B 1/2 for Flavin-tryptophan Radical
Pairs in Cryptochromes. Phys. Chem. Chem. Phys..

[51] Neil S. R. T. (2014). Broadband Cavity-enhanced
Detection of Magnetic Field
Effects in Chemical Models of a Cryptochrome Magnetoreceptor. J. Phys. Chem. B.

[52] Schleicher E. (2005). Light-induced Reactions of *Escherichia coli* DNA
Photolyase Monitored by Fourier Transform Infrared Spectroscopy. FEBS Journal.

[53] Pan J. (2004). Excited-state Properties
of Flavin Radicals in Flavoproteins: Femtosecond
Spectroscopy of DNA Photolyase, Glucose Oxidase, and Flavodoxin. J. Phys. Chem. B.

[54] Weller A., Nolting F., Staerk H. (1983). A Quantitative Interpretation of
the Magnetic Field Effect on Hyperfine-coupling-induced Triplet Fromation
from Radical Ion Pairs. Chem. Phys. Lett..

[55] Zollitsch T. M. (2018). Magnetically Sensitive
Radical Photochemistry of Non-natural Flavoproteins. J. Am. Chem. Soc..

[56] Song S.-H. (2006). Absorption and Fluorescence Spectroscopic Characterization of Cryptochrome
3 from *Arabidopsis thaliana*. Journal of Photochemistry and Photobiology B: Biology.

[57] Kao Y.-T. (2008). Ultrafast Dynamics of
Flavins in Five Redox States. J. Am. Chem. Soc..

[58] Kattnig D. R. (2016). Chemical Amplification
of Magnetic Field Effects Relevant to Avian
Magnetoreception. Nature Chem..

[59] Evans E. W. (2015). Sensitive Fluorescence-based
Detection of Magnetic Field Effects
in Photoreactions of Flavins. Phys. Chem. Chem.
Phys..

[60] Déjean V. (2020). Detection
of Magnetic Field Effects by Confocal Microscopy. Chemical Science.

[61] Antill L. M., Beardmore J. P., Woodward J. R. (2018). Time-resolved Optical Absorption
Microspectroscopy of Magnetic Field Sensitive Flavin Photochemistry. Rev. Sci. Instrum..

[62] Islam S. D. M., Susdorf T., Penzkofer A., Hegemann P. (2003). Fluorescence Quenching
of Flavin Adenine Dinucleotide in Aqueous Solution by pH Dependent
Isomerisation and Photo-induced Electron Transfer. Chem. Phys..

[63] Ikeya N., Woodward J. R. (2021). Cellular Autofluorescence
is Magnetic Field Sensitive. Proc. Natl. Acad.
Sci. U. S. A..

[64] Uzhytchak M., Smolkova B., Frtus A., Stupakov A., Lunova M., Scollo F., Hof M., Jurkiewicz P., Sullivan G. J., Dejneka A., Lunov O. (2023). Sensitivity of Endogenous
Autofluorescence in HeLa Cells to the Application of External Magnetic
Fields. Sci. Rep..

[65] Woodward, J. R. ; Ikeya, N. Radical pair based magnetic field effects in cells: the importance of photoexcitation conditions and single cell measurements. bioRxiv, November 10, 2022. 10.1101/2022.11.09.515724.

[66] Einholz C. (2021). pH-dependence of Signaling-state
Formation in *Drosophila* Cryptochrome. Arch. Biochem. Biophys..

[67] Gould I. R., Ege D., Mattes S. L., Farid S. (1987). Return Electron Transfer within Geminate
Radical Ion pairs. Observation of the Marcus Inverted Region. J. Am. Chem. Soc..

[68] Zhang M., Wang L., Zhong D. (2017). Photolyase: Dynamics and Mechanisms
of Repair of Sun-Induced DNA Damage. Photochem.
Photobiol..

[69] Liu Z. (2011). Dynamics and Mechanism
of Cyclobutane Pyrimidine Dimer Repair by
DNA Photolyase. Proc. Natl. Acad. Sci. U. S.
A..

[70] Timmel C. R., Till U., Brocklehurst B., McLauchlan K. A., Hore P. J. (1998). Effects of Weak Magnetic Fields on Free Radical Recombination
Reactions. Mol. Phys..

[71] Cheng V. W. T. (2013). A Conserved Lysine Residue
Controls Iron–sulfur
Cluster Redox Chemistry in *Escherichia coli* Fumarate
Reductase. Biochimica et Biophysica Acta (BBA)
- Bioenergetics.

[72] Müller P., Ahmad M. (2011). Light-activated Cryptochrome Reacts
with Molecular Oxygen to Form
a Flavin–superoxide Radical Pair Consistent with Magnetoreception. J. Biol. Chem..

[73] Romero E., Gómez Castellanos J. R., Gadda G., Fraaije M. W., Mattevi A. (2018). Same Substrate, Many Reactions: Oxygen Activation in
Flavoenzymes. Chem. Rev..

[74] Chaiyen P., Fraaije M. W., Mattevi A. (2012). The Enigmatic
Reaction of Flavins
with Oxygen. Trends Biochem. Sci..

[75] Arthaut L.-D. (2017). Blue-light Induced Accumulation
of Reactive Oxygen Species is a Consequence
of the *Drosophila* Cryptochrome Photocycle. PLoS One.

[76] Player T. C., Hore P. J. (2019). Viability of Superoxide-containing
Radical Pairs as
Magnetoreceptors. J. Chem. Phys..

[77] Kattnig D. R. (2017). Radical-Pair-Based
Magnetoreception Amplified by Radical Scavenging: Resilience to Spin
Relaxation. J. Phys. Chem. B.

[78] Deviers J., Cailliez F., de la Lande A., Kattnig D. R. (2022). Anisotropic magnetic
field effects in the re-oxidation of cryptochrome in the presence
of scavenger radicals. J. Chem. Phys..

[79] Görtemaker K. (2022). Direct Interaction of
Avian Cryptochrome 4 with a Cone Specific G-Protein. Cells.

[80] Wu H., Scholten A., Einwich A., Mouritsen H., Koch K.-W. (2020). Protein-protein Interaction of the
Putative Magnetoreceptor
Cryptochrome 4 Expressed in the Avian Retina. Sci. Rep..

[81] Banerjee R. (2007). The Signaling State
of *Arabidopsis* Cryptochrome
2 Contains Flavin Semiquinone. J. Biol. Chem..

[82] Berntsson O., Rodriguez R., Henry L., Panman M. R., Hughes A. J., Einholz C., Weber S., Ihalainen J. A., Henning R., Kosheleva I., Schleicher E., Westenhoff S. (2019). Photoactivation of *Drosophila
melanogaster* Cryptochrome Through Sequential Conformational
Transitions. Sci. Adv..

[83] Wong S. Y., Wei Y., Mouritsen H., Solov’yov I. A., Hore P. J. (2021). Cryptochrome Magnetoreception:
Four Tryptophans Could be Better than Three. J. R. Soc. Interface.

