# Supplementary material for: Spectroscopic Characterization of Radical Pair Photochemistry in Nonmigratory Avian Cryptochromes: Magnetic Field Effects in GgCry4a
Source: J Am Chem Soc. 2025 Jun 30;147(28):24286–98. doi: 10.1021/jacs.4c14037 (PMC12272691; doi:10.1021/jacs.4c14037)
Supplement: Supplementary file 1 [file ja4c14037_si_001.pdf]

Spectroscopic characterisation of radical pair photochemistry in non-migratory avian cryptochromes: Magnetic field effects in *Gg*Cry4a

Jamie Gravell[‡a], Patrick D.F. Murton[‡a], Tommy L. Pitcher[a,b], Kevin B. Henbest[a], Jessica Schmidt[d], Madeline M. Buffett[c], Gabriel Moise[b], Angela S. Gehrckens[c], Daniel R. Cubbin[a,c], Ana Štuhec[a,c], Lewis M. Antill[a,c], Olivier Paré-Labrosse[c], Marco Bassetto[c], Ghazaleh Saberamoli[d], Jingjing Xu[d], Corinna Langebrake[e,f], Miriam Liedvogel[e,f], Erik Schleicher[g], Stefan Weber[g], Rabea Bartölke[*d], Henrik Mouritsen[*d,h], P. J. Hore[*c], Stuart R. Mackenzie[*a], Christiane R. Timmel[*a,b]

a Department of Chemistry, University of Oxford, Chemistry Research Laboratory, Oxford, OX1 3TA, UK

b Centre for Advanced Electron Spin Resonance (CAESR), Department of Chemistry, University of Oxford, Oxford, OX1 3QR, UK

c Department of Chemistry, University of Oxford, Physical and Theoretical Chemistry Laboratory, OX1 3QZ, UK

d AG Neurosensory Sciences/Animal Navigation, Institut für Biologie und Umweltwissenschaften, Carl-von-Ossietzky Universität Oldenburg, 26129 Oldenburg, Germany

e Institute of Avian Research 'Vogelwarte Helgoland', 26386 Wilhelmshaven, Germany

f MPRG Behavioural Genomics, MPI Evolutionary Biology, 24306 Plön, Germany

g Institut für Physikalische Chemie, Albert-Ludwigs-Universität Freiburg, 79104 Freiburg, Germany

h Research Center for Neurosensory Sciences, Carl-von-Ossietzky Universität Oldenburg, 26111 Oldenburg, Germany

Supporting Information

Methods

SM.1 - Protein Expression: The *Gg*Cry4-WT was cloned, expressed, and purified following the methodology described by Xu *et al.*[1] Mutants were generated through polymerase chain reaction (PCR) using the Q5 site-directed mutagenesis kit (New England Biolabs, Ipswich, MA, USA), correct sequences were confirmed via Sanger sequencing (LGC Genomics). Table S1 summarises the primers used for each mutant.

Certain modifications from the original protocol were introduced for enhanced efficiency and optimisation. The LB media contained 10 g L^{-1} yeast extract, and the expression time was increased to 44h instead of 22h. Induction with IPTG (isopropyl-D-1-thiogalactopyranoside) was performed at an optimised concentration of 10 µM for both the WT and mutant variants. Protein expression was conducted in BL21(DE3) *Escherichia coli* cells under dark conditions, followed by purification under dim red light using immobilised metal affinity chromatography (IMAC), as outlined in Xu *et al.*,[1] but increasing the imidazole concentration in the wash buffer from 20 mM to 50 mM. This purification step was complemented by anion exchange chromatography.

For the cloning of *At*Cry1, *Arabidopsis thaliana* RNA was gratefully provided by Sascha Laubinger (University of Oldenburg, Germany) and cDNA synthesis was accomplished using SuperScript III Reverse Transcriptase (Thermo Fisher, Waltham, MA, USA) according to the manufacturer's instructions. *At*Cry1 was amplified using CloneAmpHiFi PCR Premix (Takara Bio, Shiga, Japan) using primers listed in Table S1 and a PCR reaction consisting of a 30 s denaturation step at 98°C and 30 cycles of 98°C for 30 s, 55°C for 20 s and 72°C for 25 s, followed by a final extension of 10 min at 72°C. The purified PCR product was cloned into the pFastBacHT B vector (Thermo Fisher) using *BamH*I and *Xho*I restriction sites. Baculovirus was produced in SF9 cells (Thermo Fisher) using the Bac-to-Bac Baculovirus expression system (ThermoFisher), while Tni cells (BioTrend, Cologne, Germany) were used for protein expression. Cells from 1 L culture were sedimented by centrifugation at 3745× g and resuspended in 30 mL homogenisation buffer (50 mM Tris, pH 7.4, 300 mM NaCl, 15 mM imidazole, 10 mM β-mercaptoethanol (BME), Roche cOmplete™ EDTA-free ProteaseInhibitor Cocktail) per 10 g cell pellet. Cells were lysed with a Potter-Elvehjem-homogeniser and clarified by centrifugation at 48,384× g. Clarified cell lysates were applied to Ni-NTA agarose columns (Qiagen, Hilden, Germany), pre-equilibrated with homogenisation buffer. Bound protein was eluted with elution buffer (50 mM Tris, 300 mM NaCl, 400 mM Imidazole and 10 Mm BME; pH 7.4). *At*Cry1 was diluted 1:1 in 20 mM Tris, pH 7.4 and further purified on anion-exchange 5 mL Hitrap Q columns (Cytiva, Uppsala, Sweden), after equilibration with buffer A (20 mM Tris, 150 mM NaCl, and 10 mM β-mercaptoethanol; pH 7.4). *At*Cry1 was eluted with a gradient increasing NaCl to 0.5 M at a flow rate of 1 mL/min.

All purified proteins (*At*Cry1 and all forms of *Gg*Cry4a) were concentrated to 5–6 mg mL^{-1} and, supplemented with 20 % glycerol (v/v) and 10 mM of the reducing agent (BME) to prevent dimerisation. The samples were snap-frozen in liquid nitrogen and shipped on dry ice from the production site at Oldenburg to Oxford, where they were stored at −80 °C until they were prepared for subsequent measurements.

Protein	Mutation	Forward primer	Reverse primer
*Gg*Cry4a	**W369F**	GGGGACCTTTTCATCAGCTGGGAGG	GCGGGTCAGGAAGCAGGC
*Gg*Cry4a	**R317C**	CCTCCAGATCTGTTGGTATGAGGATG	CAGATGGGGTTCCCGGCC
*Gg*Cry4a	**E320K**	CCGTTGGTATAAGGATGCTGAGAG	ATCTGGAGGCAGATGGGG
*At*Cry1		GGGCGCCATGGGATCCATGTCTGGTTC TGTATCTGGTTGT	TACCGCATGCCTCGAGTTACCCGGTTT GTGAAAG

Table S1: *Forward and reverse primer sequences used for cloning of GgCry4a W369F, R317C, E320K and AtCry1.*

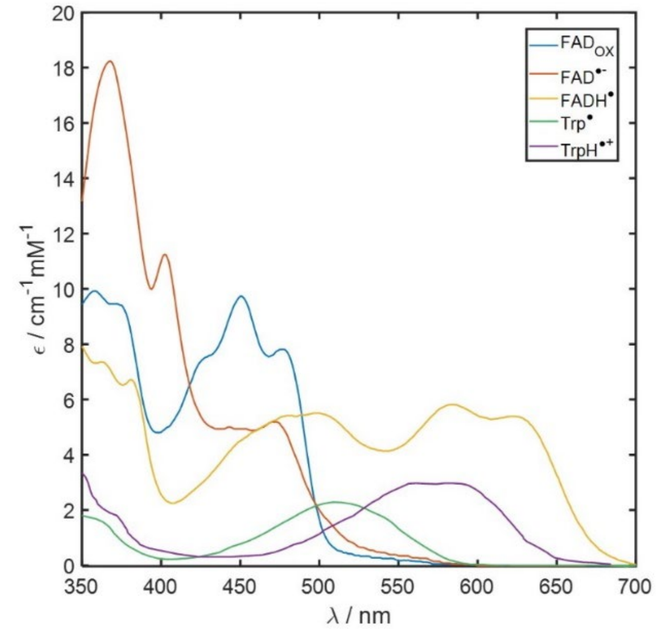

Fig. S1: Individual absorption spectra of FAD and tryptophan species involved in the cryptochrome radical pair mechanism. All spectra were taken from Ref. 8, other than that of FADH$^{\bullet}$ which is taken from Ref. 9.

***Cl*Cry4a WT (*Columba livia* / pigeon)**

TQNFTQMAGNPICLQIHWYEDAERLHKWKTAQTGFPWIDAIMTQLRQEGWIHHLARHAVACFLTRGDLWISWEEG
MKVFEELLLDADYSINAGNWMWLSA

***Gg*Cry4a WT (*Gallus gallus* / chicken)**

TPNFTKMAGNPICLQIRWYEDAERLHKWKTAQTGFPWIDAIMTQLRQEGWIHHLARHAAACFLTRGDLWISWEEG
MKVFEELLLDADYSINAGNWMWLSA

***Er*Cry4a WT (*Erithacus rubecula* / robin)**

TPNFTQMAGNPICLQICWYKDAERLHKWKMAQTGFPWIDAIMTQLRQEGWIHHLARHAVACFLTRGDLWISWEEG
MKVFEELLLDADYSINAGNWMWLSA

***Sa*Cry4a WT(*Sylvia atricapilla* / blackcap)**

TPNFTQMTGNPICLQICWYKDAERLHKWKTAQTGFPWIDAIMTQLRQEGWIHHLARHAVACFLTRGHLWISWEEG
MKVFEELLIDADYSINAGNWMWLSA

Colour coding of residues:

Trp-tetrad

317, 320

Any other differences in residue in bold and black

Fig. S2: Amino acid sequences of cryptochrome 4a (Cry4a) proteins (residues 301-400) found in the pigeon (*Cl*Cry4a WT), chicken (*Gg*Cry4a WT), European robin (*Er*Cry4a WT), and blackcap (*Sa*Cry4a WT), the latter two of which are night migratory passerines. The four W residues involved in the electron transfer cascade which has been shown to lead to radical pair formation in *Er*Cry4a WT are shown in purple.[1] All residues which are conserved in all four proteins are shown in grey while the residues in positions 317 and 320, are highlighted in red.[10] Notably, the residues in these positions are also conserved among both night migratory passerines. Other residues which vary between the Cry4a proteins of the different avian species are indicated in black.

SM.2 - Native Mass Spectrometry:

The approach adopted for the native mass spectrometry measurements was adapted from Ref. 2. Protein samples were stored at 193 K and prepared to a concentration of approximately 20 μM in a buffer containing 20 mM Tris, 20 % glycerol (v/v), pH 8 (278 K), 250 mM NaCl. For measurement, they were thawed at 278 K and exchanged into a 200 mM ammonium acetate buffer (pH 8) using a Zeba Micro Spin desalting column with a molecular weight exclusion limit of 40 kDa. Gold-plated capillaries on a Q Exactive mass spectrometer (positive ion mode) were used to electrospray (source temperature 423 K, capillary voltage 1 kV). The higher-energy C-trap dissociation (HCD) cell voltage was 5 V, with in-source trapping set to -200V, which aided the dissociation of small ion adducts.[2]

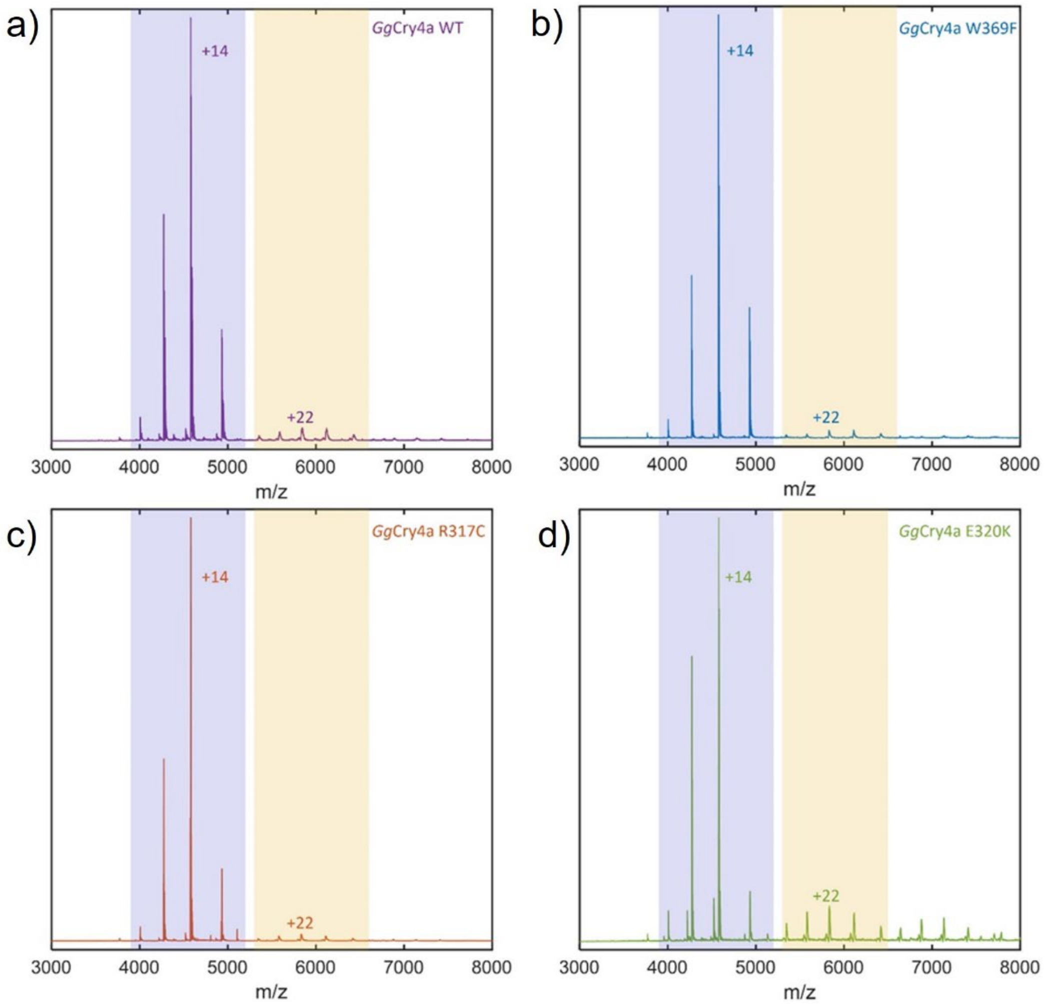

Fig. S3: Native mass spectra of *Gg*Cry4a WT (a), *Gg*Cry4a W369F (b), *Gg*Cry4a R317C (c), and *Gg*Cry4a E320K (d), plotted as relative intensities. In all four cases a charge state series consistent with the monomer was found, highlighted in light purple, as well as a charge state series consistent with the dimer, highlighted in yellow. The masses that were detected are the following: 64088.55 ± 1.02 Da (monomer *Gg*Cry4a WT; expected mass: 64219.85 Da), 128522.16 ± 30.65 Da (dimer *Gg*Cry4a WT), 64049.51 ± 0.65 Da (monomer *Gg*Cry4a W369F; expected mass: 64180.82 Da), 128250.56 ± 9.99 Da (dimer *Gg*Cry4a W369F), 64110.46 ± 2.64 Da (monomer *Gg*Cry4a R317C; expected mass: 64166.80 Da), 128270.67 ± 44.57 Da (dimer *Gg*Cry4a R317C), 64098.29±16.49 Da (monomer *Gg*Cry4a E320K; expected mass: 64218.91 Da), 128373.27 ± 47.82 Da (dimer *Gg*Cry4a E320K). All given masses refer to the holo-protein. The small peaks visible to the left of the monomer charge distribution peaks correspond to small amounts of apo-protein. The *Gg*Cry4a E320K spectrum shows some small trimer peaks right of the dimer charge state distribution.

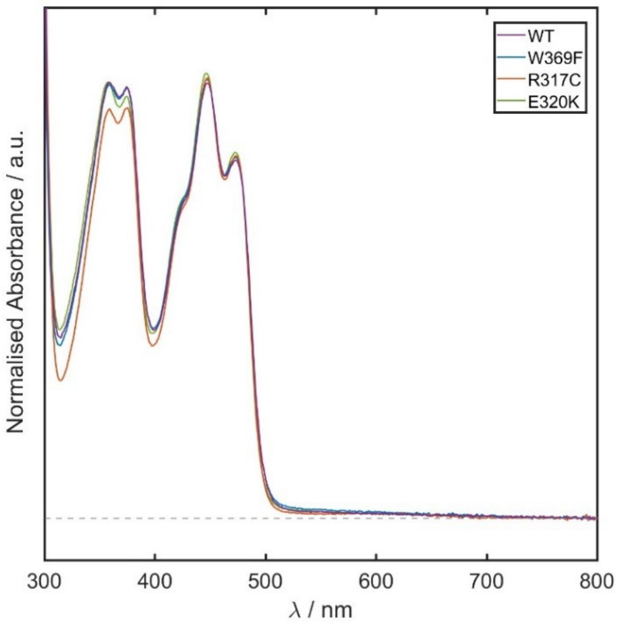

Fig. S4: UV-vis spectra of *Gg*Cry4a WT (purple), W369F (blue), R317C (red), and E320K (green) normalised at 450 nm. The fine structure of the bands below 500 nm indicates that the FAD co-factor is bound.

SM.3 - EPR:

For the EPR measurements, protein samples were prepared to a concentration of approximately 500 μM (20 % glycerol, 5 mM $K_3Fe(CN)_6$, 20 mM Tris buffer, pH 8, 250 mM NaCl) for both out-of-phase electron spin echo envelope modulation (out-of-phase ESEEM) and transient EPR (TrEPR) measurements. The buffer was air-equilibrated unless otherwise stated ([O_2] ca. 0.2 mM).

Out-of-phase ESEEM experiments were carried out at Q-band frequencies on a Bruker ELEXSYS E580 spectrometer, using a Bruker D2 resonator (EN 5107-D2), with the sample placed into quartz tubes of inner and outer diameters of 1 mm and 1.6 mm, respectively. Measurements were taken at 80 K using a nitrogen gas-flow cryostat, with the samples snap-frozen in liquid nitrogen before insertion into the resonator. Photoexcitation of the sample, at 450 nm, was provided by an OPO pumped by a Nd:YAG laser (355 nm, Opotek, Opolette). The laser light (1 mJ per pulse, 4 Hz repetition rate) was depolarised before reaching the sample.

To perform the out-of-phase ESEEM measurements, three echo-detected field swept spectra were first obtained for each sample. In each case, the first of these was obtained in the dark using the pulse sequence $(8\text{ ns})_x - \tau - (16\text{ ns})_x - \tau$ – echo, with τ = 300 ns. Any signal present at this stage was assigned to the stable FADH$^\bullet$ radical, which forms during any incidental exposure to light during sample preparation and loading into the spectrometer. The signal arising from this stable radical was used to correctly phase the out-of-phase ESEEM data being acquired, and to optimise the pulse angles towards π/2 and π radians (for the $(8\text{ ns})_x$ and $(16\text{ ns})_x$ pulses, respectively). The former was achieved by rephasing the signal such that it was all detected in one (the 'real') channel, while the latter was achieved by varying the microwave power attenuation until the detected echo intensity was maximised.

The second and third echo-detected field swept spectra were obtained using the modified pulse sequence $<16\text{ ns}>_x - T - [h\nu] - t_{\text{DAF}} - (8\text{ ns})_x - \tau - (16\text{ ns})_x - \tau$ – echo, without (dark scan) and with (light scan) the laser flash denoted by $h\nu$, respectively. Delays of T = 10 μs and t_{DAF} = 200 ns were used in each case. For *Gg*Cry4a WT, R317C, and E320K, inter-pulse delays of τ = 180 ns were used. For *Gg*Cry4a W369F, τ = 220 ns was used. In this sequence, an additional pulse $<16\text{ ns}>_x$ was included to remove any contributions from the stable radical. This was achieved by varying the microwave power of this pulse until the echo in the dark scan was minimised. Once this had been optimised, the light scan was recorded. The signal obtained in each case corresponded to an emissive-absorptive spectrum in the real channel, and an absorptive spectrum in the out-of-phase/imaginary channel. The field position of the maximum in the imaginary component of the signal was taken in each case to be the field strength used for the subsequent out-of-phase ESEEM experiment, and approximately corresponded to the zero-crossing point in the real component of the signal. All three echo-detected field swept spectra were centred at the maximum of the initial stable radical signal, with a sweep width of 20 mT. The spectra for *Gg*Cry4a R317C (in the absence of $K_3Fe(CN)_6$) are shown in Fig. S5 as a case example.

The pulse sequence $<16\text{ ns}>_x - T - h\nu - t_{\text{DAF}} - (8\text{ ns})_x - \tau - (16\text{ ns})_x - \tau$ – echo was used again for the out-of-phase ESEEM experiments. For these measurements the field strength was now fixed at a value as described above, with the inter-pulse delay τ being varied. The out-of-phase ESEEM trace in each case was generated by incrementing τ from 100 ns to 2 μs in 4 ns steps, and was recorded in the imaginary channel.

An echo-detected field swept spectrum was first obtained to determine the optimum field position at which to perform the out-of-phase ESEEM measurement, which was then detected in the imaginary channel. This field position was selected to maximise the out-of-phase (imaginary) signal, which approximately corresponds to the zero-crossing point of the emissive-absorptive signal in the real channel. Upon photoillumination, the signature of a long-lived (dark-state) radical was generated, which was assigned to the formation of FADH$^\bullet$. An additional microwave pulse was included in the sequence to minimise any contribution of this species to the above, transient light-induced signals.

The pulse sequence $<16\text{ ns}>_x - T - h\nu - t_{\text{DAF}} - (8\text{ ns})_x - \tau - (16\text{ ns})_x - \tau$ – echo was used, with T = 10 μs and t_{DAF} = 200 ns. The power of the initial $<16\text{ ns}>_x$ pulse was adjusted to minimise the dark-state signal. The out-of-phase ESEEM trace was generated by incrementing the delay τ from 100 ns to 2 μs, in 4 ns steps.

TrEPR measurements were carried out at X-band frequencies on a Bruker ELEXSYS E580 spectrometer using a Bruker MD5 resonator (ER 4118X-MD5), with a Stanford Research low noise preamplifier (SR560) utilised to improve signal-to-noise. Protein samples were loaded into quartz tubes of inner and outer diameters of 0.7 mm and 0.9 mm, respectively. These tubes were in turn loaded into larger quartz tubes to improve the quality factor. Measurements were taken at 274 K using a nitrogen gas-flow cryostat. The samples were photoexcited as described above, with a repetition rate of 1 Hz. TrEPR spectra were acquired in direct-detection mode using a transient recorder and a microwave power of 1.5 mW.

A home-written MATLAB script was used to baseline-correct the data in both the time and field domains, according to user-selected time and field points for each dataset. In the time domain, a time value corresponding to a point before the laser flash was selected. The data collected from the start of recording up to the selected time value (i.e., background data, prior to radical pair formation) was averaged for each field, to provide a time-domain offset value at each field strength. Typically, 1–2 μs of data acquired before the laser flash were used. In the field domain, four off-resonant field points were selected to determine baseline values at both low- and high-field ends of the TrEPR spectrum, with two points selected at each end. Typically, the low-field points corresponded to the first field point, 339 mT, and another point around 339.5–340 mT. The high-field points corresponded to a point around 348–348.5 mT. and the final field point, 349 mT. The data between each pair of field points were averaged to determine low-field and high-field baseline values at each time point; these two values were used to model a linear baseline across the full field range at each time point. The time-domain offset and field-domain linear baseline were then combined to an overall 2D baseline, which was subtracted from the raw data. The resultant spectra were averaged over a window of 0.5 μs following photoexcitation, and frequency-corrected to 9.637 GHz.

The below expression was used to simulate the out-of-phase ESEEM signal, $S(\tau)$, as a function of inter-pulse delay τ,

$$S(\tau) \propto \exp\left(-\frac{\tau}{T_r}\right) \int_0^{\pi} \sin\left(2\left(J - D\left(\cos^2(\theta) - \frac{1}{3}\right)\right)\tau\right) \sin(\theta)\, d\theta, \qquad \text{SE. 1}$$

where D and J are the magnitudes of the dipolar and exchange couplings (as frequencies), respectively, and T_r is the relaxation time. Each data set was simulated from 200 ns onwards, such that the effect of nuclear ESEEM modulations could be neglected.

The above expression was used as a custom function input to EasySpin's *esfit* routine, with fitting optimisation performed using the Nelder/Mead downhill simplex algorithm. Distances are determined, using a point-dipole approximation, via the following relationship,

$$D\ (MHz) = -\frac{7.80 \times 10^4}{[r\ (\text{Å})]^3}, \qquad \text{SE. 2}$$

where r is the inter-radical separation. Errors listed in Table 1 and Table S4 are the 95 % confidence intervals computed by the *esfit* routine.

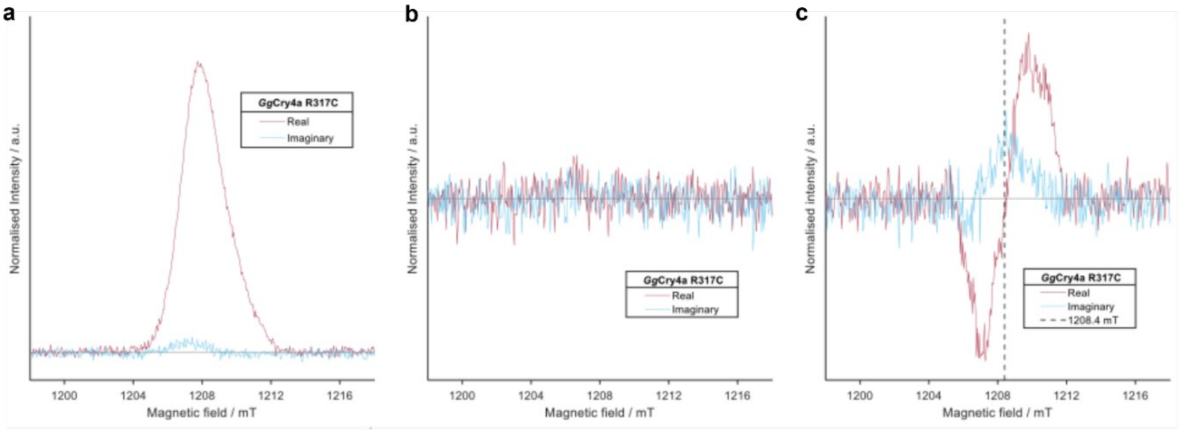

Fig. S5: Real (red) and imaginary (blue) components of echo-detected field sweeps of *Gg*Cry4a R317C in the absence of $K_3Fe(CN)_6$, recorded at 33.86 GHz. (a) Echo-detected field sweep recorded without illumination (i.e., corresponding to the long-lived FADH$^{\bullet}$ radical), using the pulse sequence $(8\ ns)_x - \tau - (16\ ns)_x - \tau - echo$, with τ = 300 ns. The 8 and 16 ns microwave pulses were optimised towards flip angles of π/2 and π radians, respectively, by attenuating the microwave power to the value that maximised the echo intensity. (b) Echo-detected field sweep as in (a), with τ = 180 ns, and an additional $<16\ ns>_x$ pulse inserted 10.2 µs before the $(8\ ns)_x$ pulse. The power of the $<16\ ns>_x$ pulse was independently adjusted to minimise any contribution from the stable radical signal observed in (a). (c) Echo-detected field sweep as in (b), with a laser flash (450 nm, 1 mJ) occurring 10 µs after the $<16\ ns>_x$ pulse and 200 ns before the $(8\ ns)_x$ pulse. The signal presented here belongs to the radical pair. (b) and (c) are recorded with an equal number of scans, with each scaled by the same normalisation constant (for the real component in (c)) to clearly illustrate the radical pair spectrum (c) that is absent without photoexcitation (b) . The field strength at which to record out-of-phase ESEEM is determined by the maximum in the imaginary component of the radical pair field sweep in (c), which approximately corresponds to the zero-crossing point of the real component of the signal. The field strength for out-of-phase ESEEM was determined to be 1208.4 mT in this example, illustrated by a dashed black line. 1208.4 mT at this microwave frequency corresponds to a g-value of approximately 2.002. At 9.637 GHz – the X-band frequency of TrEPR spectra presented in this work – the value g = 2.002 translates to a field strength of 343.9 mT, close to the maximum of the X-band TrEPR spectrum.

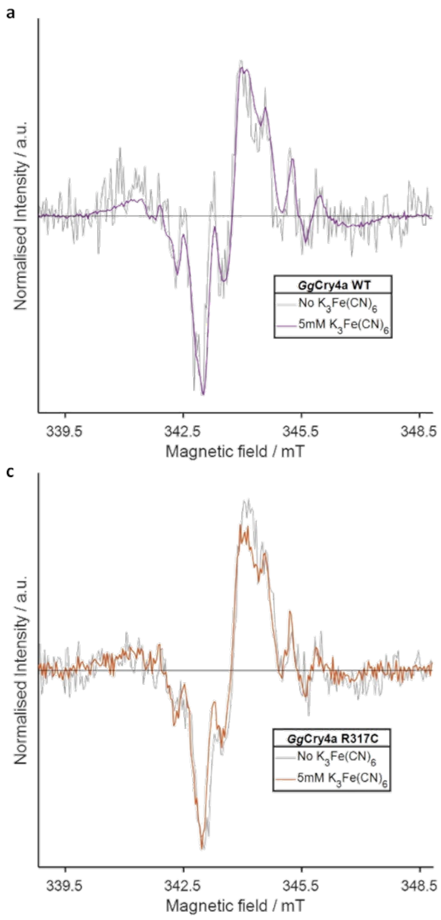

Fig. S6: Comparison between the TrEPR spectra obtained in the presence (coloured) and absence (grey) of $K_3Fe(CN)_6$. The spectra obtained in the absence of this re-oxidant have lower signal-to-noise due to photoreduction of FAD_{ox} to long-lived $FADH^{\bullet}$, and $FADH^{-}$ which are inefficiently recycled under these illumination conditions in an air-equilibrated buffer. This limits the amount of time the signal can be averaged, lowering the signal-to-noise. However, as the line shapes and fine structure in the TrEPR spectra of all four proteins agree well under both conditions, it can be concluded that the presence of $K_3Fe(CN)_6$ has little impact on the conclusions drawn from the simulation of these spectra.

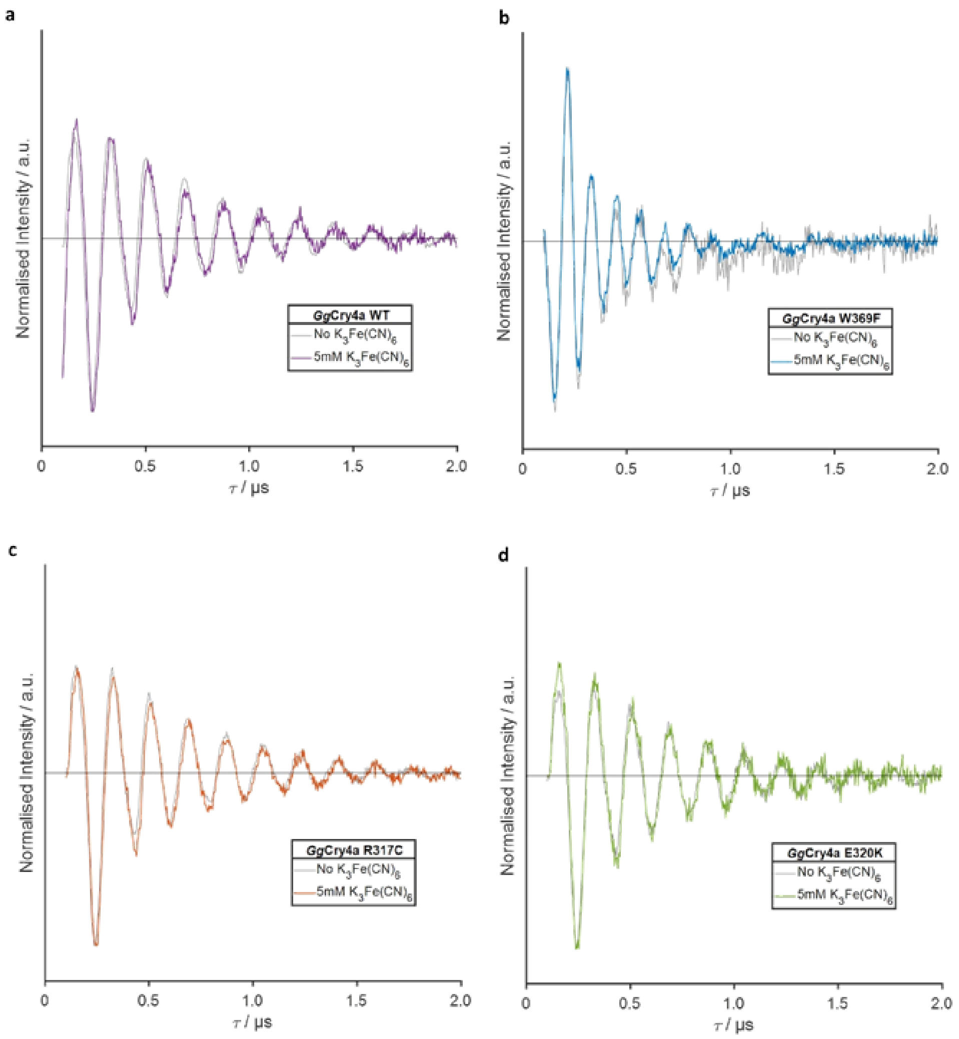

Fig. S7: Comparison between the out-of-phase ESEEM traces obtained in the presence (coloured) and absence (grey) of $K_3Fe(CN)_6$. Again, the data obtained under both conditions are in agreement and indicate that the presence of $K_3Fe(CN)_6$ has negligible effect on the inter-radical separations determined through the simulation of these experiments.

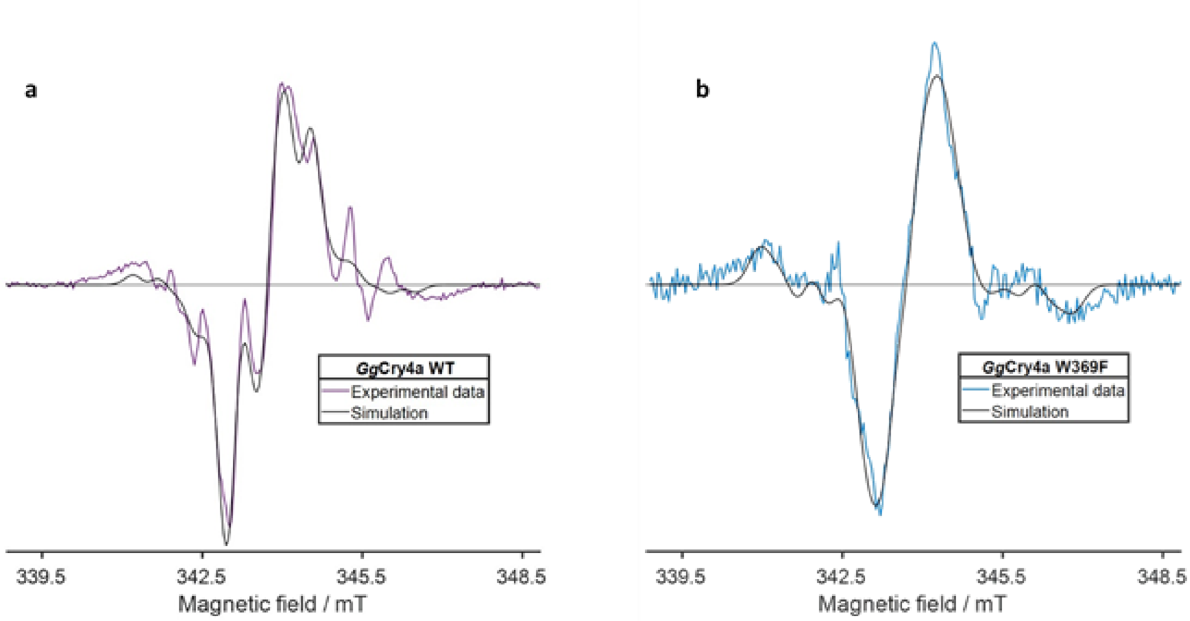

Fig. S8: Simulations (black) of the TrEPR spectra of *Gg*Cry4a WT (a) and *Gg*Cry4a W369F (b). In short, a home-written MATLAB script has been created to simulate TrEPR data of spin-correlated radical pairs (SCRPs) using a stick-spectrum approach. The model for the four-stick spectrum expected for the simplest singlet-born SCRP (illustrated in Ref. 11) is expanded upon to introduce further 'sticks' (transitions) to account for (an)isotropic hyperfine couplings, *g*-tensor anisotropies, and orientational averaging, as well as to incorporate the effects of line broadening and relative positions / orientations of the two radicals in the radical pair with respect to one another. Further details of the simulation routine are being prepared for publication elsewhere. Parameters obtained from these simulations are presented in Table S2.

	*Gg*Cry4a WT	*Gg*Cry4a W369F
g_f	[2.00439, 2.00371, 2.00218]	[2.00439, 2.00371, 2.00218]
g_w	[2.00444, 2.00336, 2.00265]	[2.00350, 2.00250, 2.00220]
$A(^{14}N)_{f,z}$ / mT	1.9	1.7
$A(^{14}N)_{f,z}$ / mT	0.42	1.0
$A(^{1}H)_{f,iso}$ / mT	0.58	0.41
$A(^{14}N)_{w,z}$ / mT	0.42	0.58
$A(^{1}H)_{w,iso}$ / mT	0.58	0.37
$[\alpha, \beta, \gamma]_w$	[92, 72, –109] °	[109, 146, –74] °
$[\theta, \varphi]_D$	[–65, 80] °	[–52, 85] °
$[D, J]$ / MHz	[–8.4, 0.8]	[–14, 0.3]
$[lw_f, lw_w]$ / G	[4.0, 4.0]	[4.2, 4.3]

Table S2: Least-squares optimised parameters for the simulation of *Gg*Cry4a WT and *Gg*Cry4a W369F TrEPR spectra. The initial *g*-tensor principal values and hyperfine coupling parameters used in the fit are taken from the literature, and based on $FAD^{\bullet-}$ and $TrpH^{\bullet+}$ radicals.[11–15] $[\alpha, \beta, \gamma]_w$ represents a set of Euler angles that define the relative orientations of the two radicals. These are defined as a ZYZ sequence of rotations that converts the g-tensor axes of $FAD^{\bullet-}$ into those of the relevant $TrpH^{\bullet+}$ radical. $[\theta, \varphi]_D$ are the declination and azimuth angles, respectively, that define the dipolar vector between radicals with respect to the g_z axis of $FAD^{\bullet-}$. Both angle sets are based on values obtained from a *Gg*Cry4a WT homology model, modelled from the crystal structure of *Cl*Cry4a (PDB: 6PU0).[16] The strength of the dipolar coupling in each simulation is fixed to that found from out-of-phase ESEEM. The exchange coupling values are presented here as fitted parameters, but due to their relatively small magnitude and the number of other parameters involved in TrEPR simulation are more accurately determined via out-of-phase ESEEM. Gaussian line broadening effects on the spectrum from both $FAD^{\bullet-}$ and $TrpH^{\bullet+}$ contributions are introduced into the simulation via lw_f and lw_w, respectively.

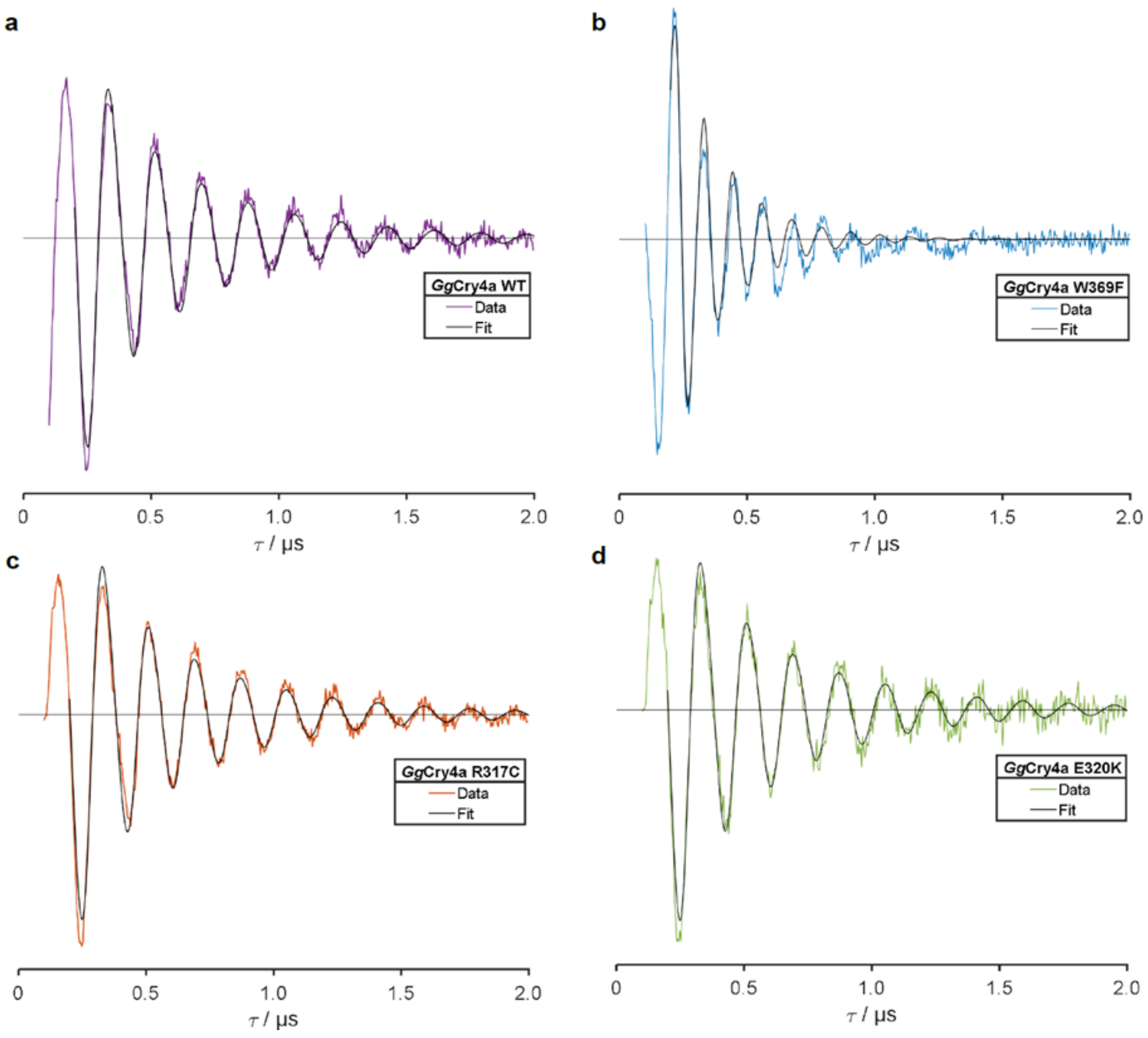

Fig. S9: Out-of-phase ESEEM traces for *Gg*Cry4a WT (a), W369F (b), R317C (c) and E320K (d), obtained with 5 mM $K_3Fe(CN)_6$. These are reproductions of the data shown in Fig. 2c,d in the main text, along with simulations based on equation SE.1 (fitting from 200 ns onwards), presented in black for each protein. Parameters extracted from the fit are found in Tables 1 and Table S4.

FAD-X	Inter-residue separation / Å
X = W395	7.7
X = W372	13.1
X = W318	17.7
X = W369	21.4
X = Y319	24.5

Table S3*:* Centre-to-centre separations between the FAD co-factor and various residues for a *Gg*Cry4a homology model, based on the crystal structure of *Cl*Cry4a WT.

Protein	J / MHz	T_r / µs
*Gg*Cry4a WT	0.03 ± 0.02	0.60 ± 0.04
*Gg*Cry4a E320K	0.05 ± 0.02	0.63 ± 0.05
*Gg*Cry4a R317C	0.03 ± 0.04	0.61 ± 0.02
*Gg*Cry4a W369F	0.31 ± 0.06	0.25 ± 0.05

Table S4*:* Exchange coupling (J) and relaxation times (T_r) obtained from least-squares fitting to out-of-phase ESEEM traces shown in Fig. 2c-d. See EPR methods section above.

SM.4 – TA:

For the transient absorption (TA) spectroscopy measurements, protein samples were prepared to a concentration of approximately 65 µM in a buffer containing 20 mM Tris, 20 % glycerol (v/v), pH 8 (278 K), 250 mM NaCl. These samples were air-equilibrated ([O_2] ca. 0.2 mM), and no external oxidant was added.

The experimental protocol used for the acquisition of TA spectroscopy data, obtained using an Ultrafast Systems EOS spectrometer, followed closely that reported in the supporting information of Ref. 1. Briefly, the sample was photoexcited at 450 nm (30 µJ per pulse, 80 ps pulse width, repetition rate 1 kHz) by a mode-locked Nd:YAG laser (355 nm) pumping an OPG (Ekspla PL2210 and PG403). A supercontinuum light source provided a probing range of 350-900 nm (repetition rate 2 kHz, 1 ns pulse width). The samples were cooled to 278 K, by an Oxford Instruments cryostat. An external magnetic field (25 mT) was provided by a set of home-built Helmholtz coils, controlled by a custom written LabVIEW programme.

Magnetic field effects were obtained by acquiring the TA data in the presence ($\Delta A(B_0)$) or absence ($\Delta A(B_0 = 0)$) of a 25 mT magnetic field. Each acquisition period (field on or field off measurement) lasted 15 s, with a 3 min interval between each successive measurement. Such measurements were conducted in a sequence of (field) on-off-off-on cycles to minimise the effects of photodegradation on the resulting data. The sample was, therefore, illuminated for a total time of 60 s during each cycle. The measurements shown here are the result of averaging 120 such cycles together and subsequently background correcting, wavelength and time-averaging the TA data to yield kinetic traces and time-resolved spectra. Data processing was performed using a custom written LabVIEW code. Further data analysis, including kinetic fitting and magnetic field effect determination, was conducted using home-written MATLAB programs.

The kinetic data, shown in Fig. 3a were obtained in the absence of an applied field and were fit to a bi-exponential function including a constant offset,

$$M_1(t|a_1, a_2, a_3, \tau_1, \tau_2) = a_1 \exp\left(-\frac{t}{\tau_1}\right) + a_2 \exp\left(-\frac{t}{\tau_2}\right) + a_3 \qquad \text{SE. 3}$$

constant offset, The uncertainties quoted in the manuscript in Table 2 are dominated for τ_1 by the probe pulse duration (1ns) and for τ_2 calculated by employing the inverse Hessian method.

The magnetic field effects, shown in Fig. 4b, were calculated using

$$\Delta\Delta A = \Delta A(B_0) - \Delta A(B_0 = 0) \qquad \text{SE. 4}$$

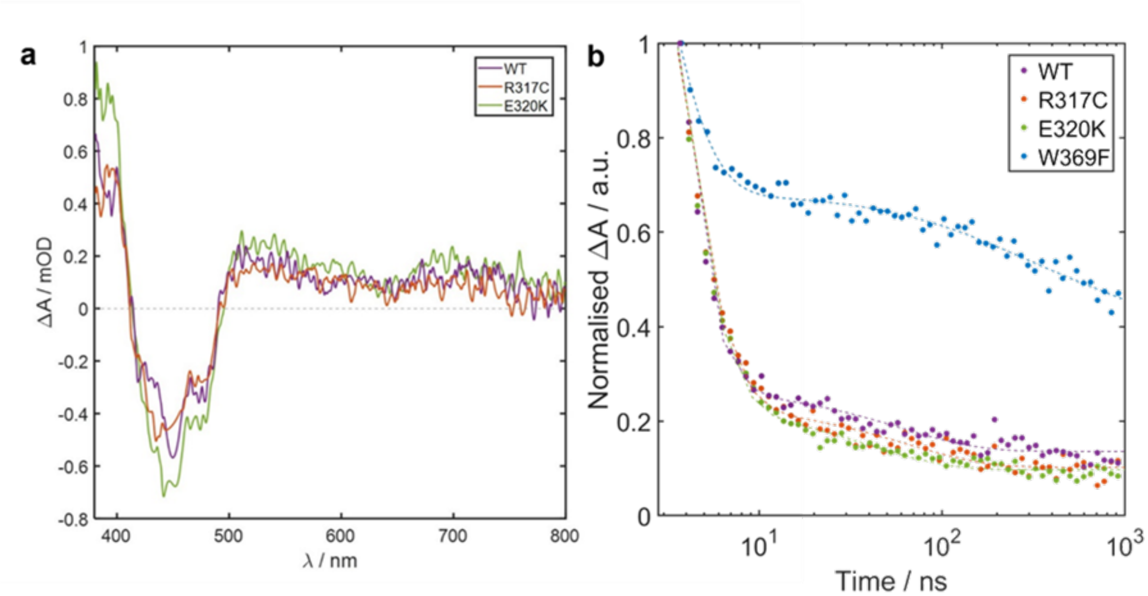

Fig. S10: a) ΔA spectra, time-averaged over10-13 ns after the pump pulse, obtained for *Gg*Cry4a WT (purple), R317C (red), and E320K (green). The positive ΔA over the 500-650 nm region indicates radical pair generation with spectral shapes consistent with $FAD^{\bullet-}$ and $TrpH^{\bullet+}$.[17] The absorbance centred at 700 nm is assigned to $^3FAD^*$ resulting from the intersystem crossing of $^1FAD^*$ in a (minor) fraction of the protein sample which is misfolded resulting in a dramatic reduction in the rate of electron transfer between FAD and W395 (W_A). b) As in Fig. 4a), presented with a logarithmic time axis, to better visualise the early component of the kinetic decays.

Spectral Region / nm	τ_1 / ns	τ_2 / ns
370-400	1.6 ± 0.5	275 ± 12
460-490	0.9 ± 0.5	193 ± 4
600-650	2.4 ± 0.5	270 ± 7

Table S5*:* Lifetimes obtained through bi-exponential fitting of the kinetic data presented in Fig. S10.

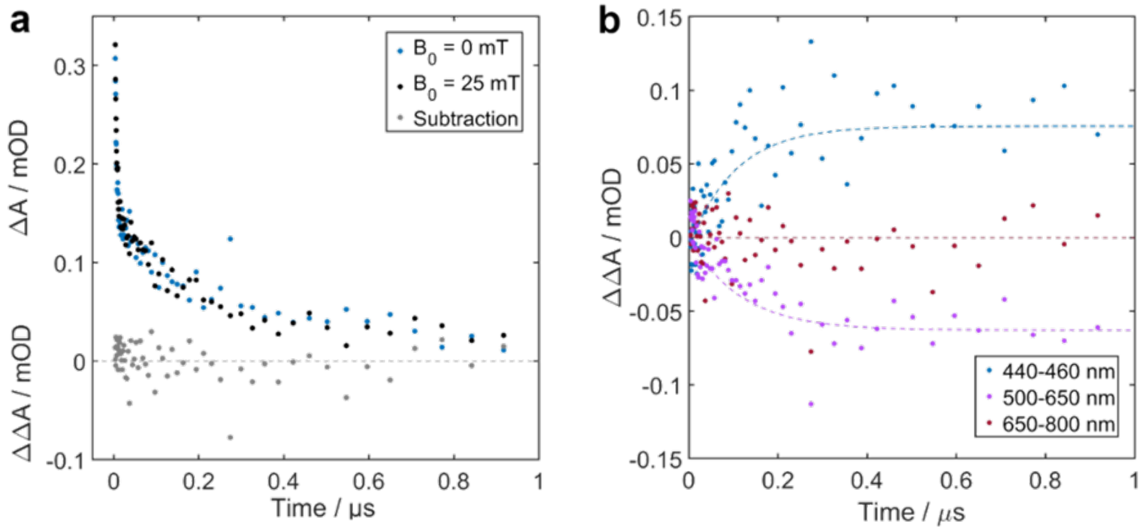

Fig. S11: (a) ΔA(t) traces of air-equilibrated GgCry4a W369F, averaged over 650-800 nm, obtained in the absence (blue) and presence (black) of a 25 mT external magnetic field. The $\Delta\Delta A(t, B_0)$ obtained from these data using Eq. (1) (see main text) is shown in grey, below the ΔA(t) data. No $\Delta\Delta A(t, B_0)$ is detected in this region. (b) Comparison of the $\Delta\Delta A(t, B_0)$ data of GgCry4a W369F obtained in the 440-460 nm (blue), 500-650 nm (purple) and 650-800 nm (maroon) spectral regions. Note the sign change between the ground state bleach (440-460 nm, positive) and radical region (500-650 nm, negative) and the lack of magnetic field effect between 650 and 800 nm. The dashed lines are mono-exponential fits to the data (blue, purple) or a line to guide the eye (maroon).

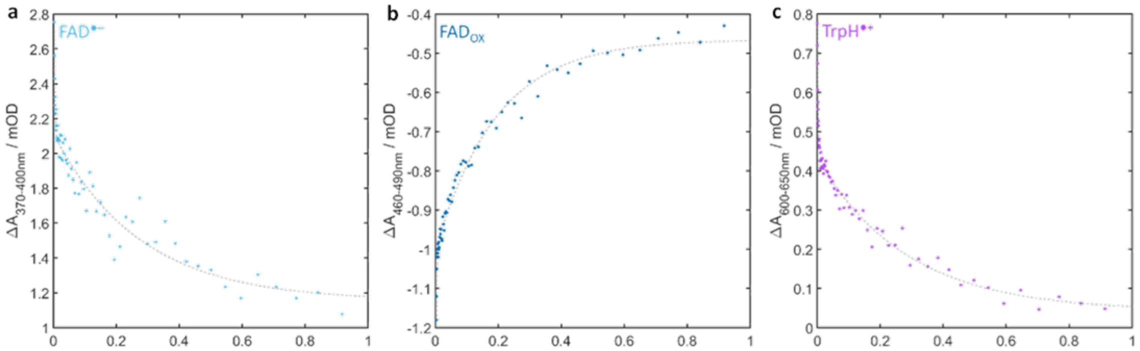

Fig. S12*: ΔA* signatures of *Gg*Cry4a W369F averaged over the 370-400 nm (a), 460-490 nm (b), and 600-650 nm (c) spectral regions accompanied by a bi-exponential fit (black line dashed lines) including a constant offset. The predominant absorber in each spectral region is highlighted in the corner of the corresponding figure.

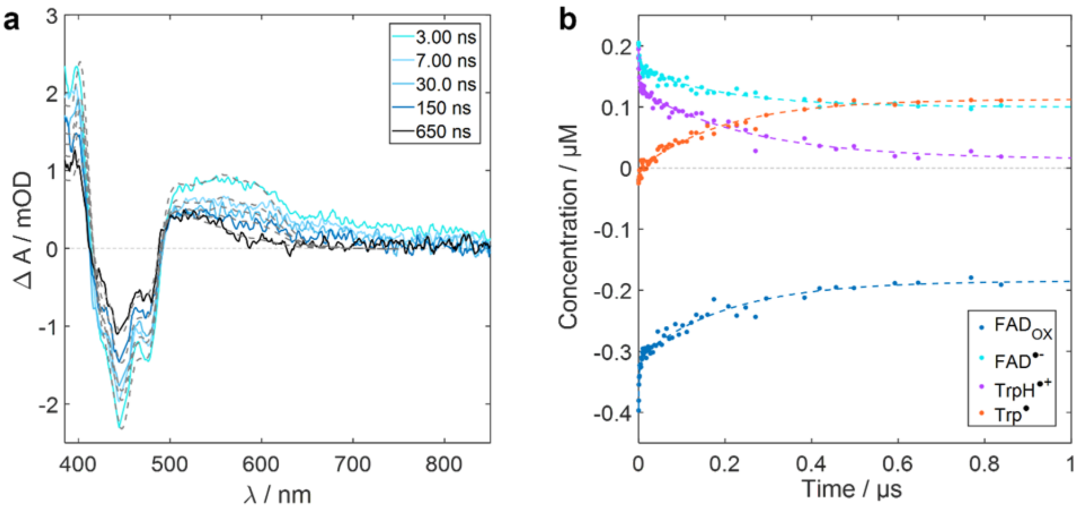

Fig. S13: a) GgCry4a W369F global analysis spectral fit results (grey dotted lines) and ΔA spectra (blue lines) at several time points. Fits were made using the individual species spectra of TrpH$^{•+}$, Trp$^{•}$, FAD$^{•-}$ and FAD$_{OX}$ shown in Fig. S1**.** Analysis generally fits the data well. b) GgCry4a W369F individual species kinetics, extracted from the global fit in a), showing the formation of Trp$^{•}$ from TrpH$^{•+}$ and the corresponding partial decay of FAD$^{•-}$ and GSB recovery. Note the matching persistent concentrations of Trp$^{•}$ and FAD$^{•-}$. Bi-exponential fits to the globally extracted time-evolution in the concentrations of all four species are given by the correspondingly coloured dashed lines.

Species	τ_1 / ns	τ_2 / ns
FAD$^{•-}$	1.9 ± 0.5	201 ± 26.4
FAD$_{OX}$	1.9 ± 0.5	204 ± 20.8
TrpH$^{•+}$	2.0 ± 0.5	260 ± 27.9
Trp$^{•}$	1.9 ± 0.5	198 ± 17.3

Table S6: Bi-exponential fits to the individual species kinetics extracted by global analysis (Fig. S12). All species evolve on a similar timescale and broadly agree with the fits at various spectral regions shown in Table S5. As discussed in the main text, this behaviour indicates a similar singlet recombination and TrpH$^{•+}$ deprotonation rate.

SM.5 - BBCEAS:

All BBCEAS protein samples were prepared to a concentration of approximately 30 μM in a buffer consisting of 20 mM Tris, 20 % glycerol (v/v), pH 8 (278 K), 250 mM NaCl and were run at 278 K under an oxygen atmosphere (applied by an O_2 filled balloon, *ca* 1.08 x 10^5 Pa and *ca.* 0.8 – 0.9 mM in 20% glycerol v/v). The BBCEAS general experimental protocol and working principles have already been outlined in Refs. 1 and 3.[3] Briefly, 180 μL of sample was placed in a quartz sample cell (Hellma analytics, QS 165-40, 1 mm path length and cooling jacket) positioned in the centre of a 20 cm optical cavity. The optical cavity was formed of two opposite facing broadband mirrors (Layertec GmbH) with mean reflectivities of R > 0.9978 (400 nm - 800 nm). Samples were photoexcited by a 450 nm continuous-wave diode laser (Oxxius LBX-450) which was focused into the sample at a sharp angle relative to the cavity axis such that coupling into the cavity was minimised. Simultaneously, the optical cavity, and thus sample, was probed by a pseudo-continuous white light super-continuum (SC) source (NKT SuperK Extreme, 78 MHz with 700 ps pulses). The light exiting the optical cavity was subsequently passed through a spectrograph (Andor Shamrock SR303i) and detected by a CCD camera (Andor Newton). Magnetic fields were applied from a set a custom-built Helmholtz coils (up to ± 30 mT). A custom-written LabVIEW program controlled the experiment and logged acquired data.

For the protein experiments presented here, samples were photoexcited continuously with *ca.* 3.82 kW m^{-2} (3 mW, *ca.* 1 mm beam diameter) over a period of 10 s. Concurrently, the cavity and sample were probed continuously by the SC source with the light transmitted through the cavity detected by the CCD with an exposure time, and thus time resolution, of 1 ms. During the photoexcitation cycle, a magnetic field was symmetrically modulated on and off with a period of 200 ms. Varying field strengths and polarities were applied randomly from the set of desired field values with a different magnetic field applied for every 10 s illumination cycle. After photoexcitation, the sample was allowed to re-oxidise in the dark for 60 min. A ground state spectrum was acquired before every photoexcitation period.

Data analysis was carried out using a custom written MATLAB program. In general, cavity transmission signals, acquired *via* the CCD, are converted into absorption (A) signals with the following expression

$$A(t,\lambda) = -\log\left(\frac{R(\lambda)_{eff}^2 - 1 + \sqrt{1 + 2R(\lambda)_{eff}^2\left(2\left(\frac{I(t,\lambda)}{I_0}\right)^2 - 1\right) + R(\lambda)_{eff}^4}}{2R(\lambda)_{eff}^2\frac{I(t,\lambda)}{I_0(\lambda)}}\right) \qquad \text{SE. 5}$$

where $I(t,\lambda)$ is the light exiting the cavity continuously detected by the CCD, $I_0(\lambda)$ is the light intensity from a blank (water) reference cavity and $R(\lambda)_{eff}$ is the effective reflectivity which accounts for non-sample cavity transmission losses (e.g. scattering). The $R(\lambda)_{eff}$ was determined following calibration of light transmission through the optical cavity from a series of dye concentrations. The details of this calibration procedure may be found elsewhere (Refs. 3 and 4). Finally, photoinduced absorption (ΔA) and MFEs ($\Delta\Delta A$) were calculated as follows,

$$\Delta A = A_{hv} - A_{GS}\,, \qquad \text{SE. 6 a}$$

$$\Delta\Delta A = \Delta A(B_0) - \Delta A(B_0 = 0)\,, \qquad \text{SE. 6 b}$$

where A_{hv} is the absorption with photoexcitation, A_{GS} is the ground state absorbance (without excitation) and $\Delta A(B_0)$ and $\Delta A(B_0 = 0)$ are the photoinduced absorption with and without an applied magnetic field respectively. $B_{1/2}$ values (Table S6) were estimated from the MARY plots shown in Fig. 6b by least-squares fitting the data to the following Lorentzian curve ($\mathcal{L}$)

$$\mathcal{L}(B_0|a_1,a_2) = a_1\frac{a_2^2}{{B_0}^2 + a_2^2} - a_1 \qquad \text{SE. 7}$$

where B_0 is the strength of the applied magnetic field, a_1 determines the saturation point of the $\Delta\Delta A$ and a_2 is the $B_{1/2}$. Errors in the fit were estimated using the inverse Hessian method.

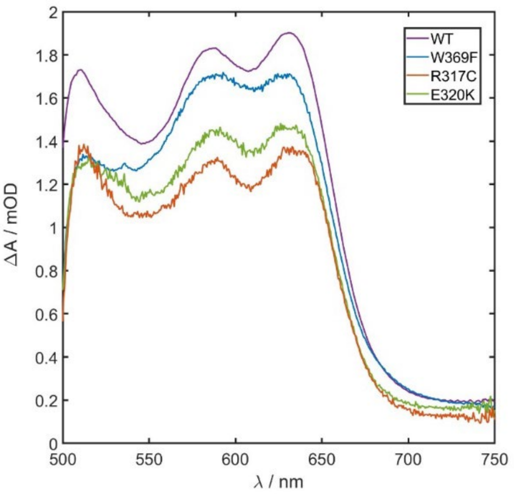

Fig. S14: Δ*A* spectra of GgCry4a WT (purple), W369F (blue), R317C (red), and E320K (green) obtained 9 s after the onset of blue light photoexcitation.

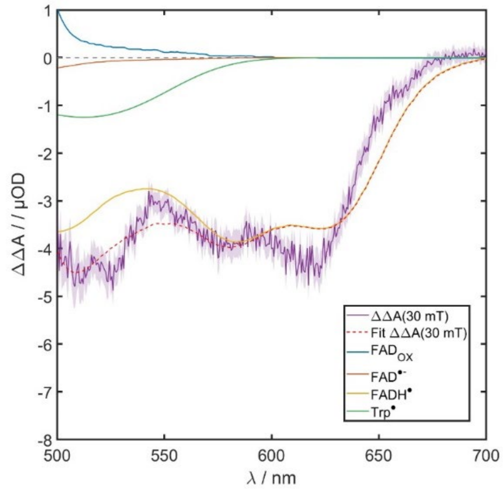

Fig. S15: ΔΔ*A* spectrum for *Gg*Cry4a WT, obtained using an applied magnetic field strength of 30 mT. The spectrum can be successfully reproduced (red dashed line) using the absorbance profiles of FAD_{ox}, $FAD^{\bullet -}$, $FADH^{\bullet}$, and $Trp^{\bullet}$, from S1, as basis spectra.

Protein	$B_{1/2}$ / mT
*Gg*Cry4a WT	3.82 ± 0.12
*Gg*Cry4a W369F	3.71 ± 0.10
*Gg*Cry4a R317C	4.52 ± 0.22
*Gg*Cry4a E320K	4.77 ± 0.21

Table S7: $B_{1/2}$ parameters obtained by least-squares fitting of a Lorentzian lineshape to the MARY curves shown in Fig. 6b. See SM.5 for more details.

SM.6 - Confocal Microscopy:

The experimental procedure and data analysis protocols for the confocal microscopy experiments were adapted from Ref. 5. The protein samples were prepared to a concentration of approximately 50 μM in a buffer containing 20 mM Tris, 20 % glycerol (v/v), pH 8 (278 K), 250 mM NaCl. A 20 μL droplet of this solution was pipetted onto a glass-bottom dish (Fisher Scientific) and subsequently surrounded by (but not in direct contact with) 200 μL of the above buffer solution, to prevent sample evaporation during data acquisition. The sample temperature was maintained at 278 K during the experiment using a custom-built aluminium cooling stage. An external magnetic field (17 mT) was provided by a pair of solenoids wound around ferrite cores (rise time 70 ms). To allow re-oxidation of the protein samples during data acquisition, a 3D-printed rubber seal, adapted with an inlet and outlet for oxygen flow was used to enclose the glass bottom dish housing the sample. The oxygen gas was hydrated prior to contact with the sample by passing it through a Dreschel bottle partially filled with deionised water, to prevent sample dehydration. The sample was exposed to oxygen flow in this manner for 16 h prior to measurement to allow saturation of the buffer solution. Oxygen flow was maintained during the measurements. The concentration of oxygen in the solution is estimated as 0.8-0.9 mM.

The Leica SP8 confocal microscope used for these measurements allowed for synchronised triggering of the magnetic field coils, by an arbitrary waveform generator (Rigol DG1022), and fluorescence acquisition by the commercially written programme. Laser excitation (448 nm, 70 μW) was provided from $t = 0$ by a diode laser focused onto the sample by a 63x objective lens (NA 1.4) and raster scanned across the field of view. The fluorescence was averaged over the entire region in which $^1FAD^*$ is known to emit (475-650 nm). The field was turned on (17 mT) and off (0 mT) sequentially during the measurement leading to modulations in the fluorescence intensity in synchrony with the magnetic field switching. The field switching period (95.926 s) was selected as it was an integer multiple of the frame acquisition time (959.26 ms) and allowed for full profiling of the temporal dynamics observed within a field on-off cycle. The fluorescence of the entire field-of-view (100 x 100 μm^2) was averaged to obtain a fluorescence time trace (e.g. as shown in Fig. 6c for *Gg*Cry4a WT). After background subtraction (See Ref. 5 and Fig. S15) of this data a mean %MFE (discarding the first and last field on-off cycles due to poor centreline fitting) as shown in Fig. 6d, was calculated as

$$\%\text{MFE} = \frac{I_F(B_0) - I_F(B_0 = 0)}{I_F(B_0 = 0)} \times 100 \qquad \text{SE. 8}$$

The errors shown in Fig. 6d are the standard error of the mean of the field on-off cycles used to calculate the mean %MFE. The dynamics observed during one field on-off cycle were fit to a single exponential function, as in Refs. 5, 6. The errors in these lifetimes, quoted in Table 3, are bootstrapped estimates obtained with 400 random samples.

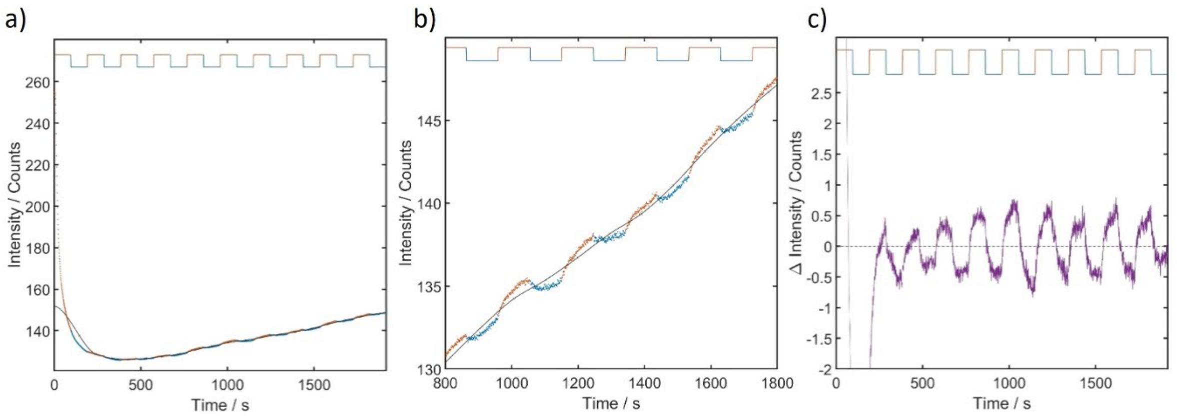

Fig. S16: a) Time-evolution of the fluorescence of *Gg*Cry4a WT, as shown in Fig. 6c, including the interpolant (black) fit to the centre points between each field-on (red) and field-off (blue) fluorescence segment. See Ref. 5 for further details of the data analysis method. b) Same as a) between 800-1800 s to more clearly illustrate the background fit and dynamics within each field-on and field-off fluorescence segment. c) Background subtracted time evolution of the fluorescence intensity of GgCry4a WT (purple). As noted in Ref. 5, the fit to this type of data is poor when the fluorescence is rapidly changing, as occurs at the beginning of the experiment, and at the final MFE step in the experimental data. Consequently, the mean %MFE for all four proteins, shown in Fig. 6d is calculated by averaging from the third to the ninth field-on/field-off cycle.

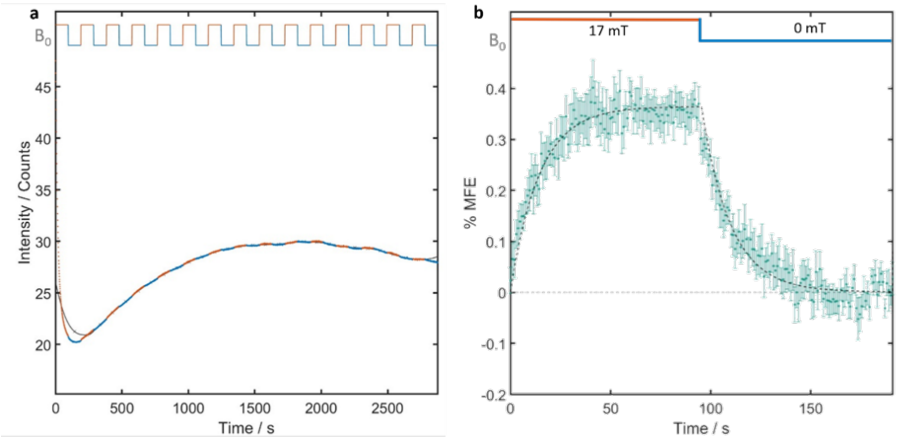

Fig. S17: a) Time evolution of the fluorescence intensity of a solution of *At*Cry1 in 10 mM Tris buffer at pH 7.4 (278 K) with 150 mM NaCl, and 30 % glycerol (v/v). This buffer was chosen for consistency with previous measurements.[6] A magnetic field is switched between 0 and 17 mT, with a period of 191.8 s during the acquisition, as in Fig. 6c. b) Time-averaged %MFE, calculated from Eq. 2, as detailed in SM.6. Due to poor centreline fitting the first two and last field on-off cycles were discarded during the averaging procedure. The time-constant, obtained by exponential fitting was τ_{eMFE} = 16.65 $\pm$ 1.39 s, significantly longer than the rise-time of 3.4 $\pm$ 0.9 s reported in Ref. 6.

SM.7 - Continuous Illumination:

Protein samples were prepared to a concentration of approximately 25 µM in a buffer containing 20 mM Tris, 20 % glycerol (v/v), pH 8 (298 K), 250 mM NaCl. These samples were air-equilibrated ([O_2] ca. 0.2 mM), and no external oxidant or reductant was added. A Carey 60 UV-vis spectrophotometer was used to monitor the (dark) reversion of long-lived photoproducts to their ground state. Radicals were generated by 60 s of illumination with a 450 nm LED (25 W m^{-2}). Spectra (300-800 nm) were recorded at 75 s intervals after illumination had ceased. The traces shown in Fig. 7b were obtained by averaging the absorbance at each time point over 605-615 nm, a region where the only absorber on the minute timescales probed here is $FADH^{\bullet}$. The lifetimes quoted in Table 4 are obtained by fitting a mono-exponential function including a constant offset,

$$M_2(t|a_1, a_2, \tau_1) = a_1 \exp\left(-\frac{t}{\tau_1}\right) + a_2 \qquad \text{SE. 9}$$

The errors quoted are the errors in the fit, estimated from the inverse Hessian method.

Protein	τ_{rec} / min
*Gg*Cry4a WT	66.52
*Gg*Cry4a R317C	67.57
*Gg*Cry4a E320K	36.73
*Gg*Cry4a W369F	35.98

Table S8: Recovery lifetimes of $FADH^{\bullet}$ obtained using global analysis of the irradiation recovery experiments, as described in SM.8, and for which representative data and spectral fits are shown for *Gg*Cry4a WT in Fig. 7a. The analysis assumes a first-order recovery of $FADH^{\bullet} \rightarrow FAD_{ox}$.

SM.8 - Global Target Analysis:

Global target analysis was utilised in the analysis of the BBCEAS data (Fig. 5a inset**,** Fig. S14), TA data (Fig. S12) and the continuous illumination data (Fig. 7a.). In general, a 2D absorption (or ΔA) data array, $\boldsymbol{D}$, with dimensions of wavelength, λ , by time, t, can be factorised such that

$$\boldsymbol{D}(\lambda, t) = \boldsymbol{C}(t)\boldsymbol{S}(\lambda)^{\mathrm{T}} = \sum_{i=1}^{n} c(t)_i s(\lambda)_i, \qquad \text{SE. 10}$$

where $\boldsymbol{C}(t)$ is a *m* x *n* matrix of coefficients proportional to concentration, $\boldsymbol{S}(\lambda)$ is a *q* x *n* matrix of individual species absorption spectra, *m* and *q* are the number of time and wavelength points over which the data array is acquired and *n* is the number of absorbing species within the data signal (Ref. 7). Given the acquired data are known, if either the kinetic evolution, determined by $\boldsymbol{C}(t)$, or absorbing species, $\boldsymbol{S}(\lambda)$, is known (or a suitable model assumed) then the unknown factor can be calculated by solving equation SE. 10 with numerical methods. Here MATLAB's backslash function was employed (least-squares solution).

For the construction of Fig. 7a an A→B kinetic model was assumed for $\boldsymbol{C}(t)$ such that

$$\boldsymbol{C}(t)^{\mathrm{T}} = \begin{pmatrix} \xi e^{-t/\tau} \\ 1 - \xi e^{-t/\tau} \end{pmatrix}, \qquad \text{SE. 11}$$

where τ is the time constant for the formation of B from A, and ξ determines the relative amount of A and B at *t = 0*. Solutions of SE. 10 with respect to $\boldsymbol{S}(\lambda)$ were used to obtain estimates for τ and ξ and thus more accurate solutions for $\boldsymbol{S}(\lambda)$ by minimising the least square error (*Err*) in the fitted data array $\boldsymbol{D}_{fit}(\lambda, t)$ with respect to the measurement data, $\boldsymbol{D}(\lambda, t)$. That is

$$Err = (\boldsymbol{D}(\lambda, t) - \boldsymbol{D}_{fit}(\lambda, t))^2. \qquad \text{SE. 12}$$

Conversely, to extract individual species kinetics from the BBCEAS and TA ΔA data (Fig. S14 and Fig. S12, respectively), the absorbing species spectra were assumed and taken from the absorption spectra shown in Fig. S1 (Refs. 8 and 9). Specifically, due to the BBCEAS time scale and known cryptochrome photoinduced radical lifetimes, four contributing species were assumed, namely: FAD_{OX}, $FAD^{\bullet -}$, $FADH^{\bullet}$, and $Trp^{\bullet}$. With the assumed $\boldsymbol{S}(\lambda)$ matrix, SE. 10 was solved for $\boldsymbol{C}(t)$ iteratively over each time point such that SE.10 was reduced to a series of calculations of the form

$$\boldsymbol{d}_j(\lambda) = \boldsymbol{S}(\lambda)\boldsymbol{c}_j, \qquad \text{SE. 13}$$

where $\boldsymbol{d}_j(\lambda)$ is a column of $\boldsymbol{D}(\lambda, t)$ at the *j*th time point and $\boldsymbol{c}_j$ is a row of $\boldsymbol{C}(t)$ at the *j*th time point.

SM 9 – O_2 as reoxidant:

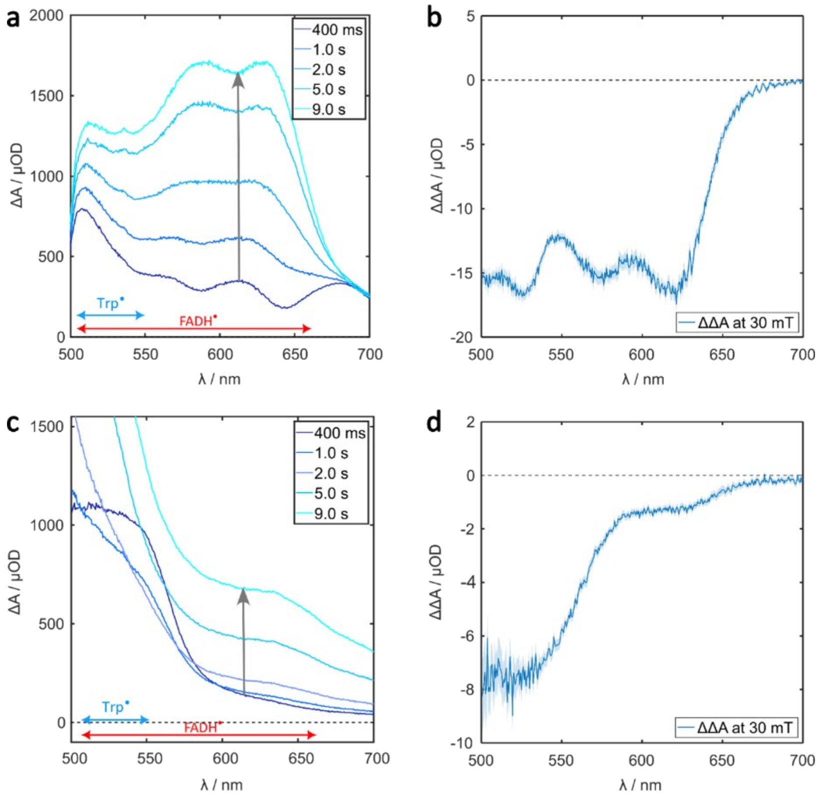

Fig. S18: a) BBCEAS Δ*A* spectra of *Gg*Cry4a W369F under an oxygen atmosphere showing predominant formation and accumulation of FADH•. b) BBCEAS time averaged *Gg*Cry4a W369F ΔΔ*A* spectrum under an oxygen atmosphere at 30 mT. The spectral shape indicates a MFE predominantly on the accumulation FADH•, reflecting the behaviour of the Δ*A* spectra in a). c) BBCEAS Δ*A* spectra of *Gg*Cry4a W369F in the presence of 5 mM potassium ferricyanide where, what appears to be, initial Trp• formation (peak centred at 525 nm at 400 ms) is followed by the formation and accumulation of some species whose absorption peaks below 500 nm. d) BBCEAS time averaged *Gg*Cry4a W369F ΔΔ*A* spectrum in the presence of 5 mM potassium ferricyanide at 30 mT. The spectral shape indicates a MFE predominantly on the accumulation of Trp• (peak centred at 525 nm) with an additional FADH• shoulder between 550 and 700 nm. Due to the optical cavity and high potassium ferricyanide absorption below 500 nm, too little light escapes the optical cavity to resolve spectral features below 500 nm. The identity of the species forming at late times in the presence of potassium ferricyanide and the influence potassium ferricyanide has on the BBCEAS results is the subject of forthcoming publications, currently in preparation.

References

1. Xu, J. *et al.* Magnetic Sensitivity of Cryptochrome 4 from a Migratory Songbird. *Nature* **594**, 535–540 (2021).
2. Hanić, M. *et al.* Dimerization of European Robin Cryptochrome 4a. *J. Phys. Chem. B* **127**, 6251–6264 (2023).
3. Neil, S. R. T. *et al.* Broadband Cavity-enhanced Detection of Magnetic Field Effects in Chemical Models of a Cryptochrome Magnetoreceptor. *J. Phys. Chem. B* **118**, 4177–4184 (2014).
4. Konowalczyk, M. High-sensitivity Measurements of Weak Magnetic Field Effects. (University of Oxford, 2020).
5. Déjean, V. *et al.* Detection of Magnetic Field Effects by Confocal Microscopy. *Chem. Sci.* **11**, 7772–7781 (2020).
6. Kattnig, D. R. *et al.* Chemical Amplification of Magnetic Field Effects Relevant to Avian Magnetoreception. *Nat. Chem.* **8**, 384–391 (2016).
7. van Stokkum, I. H. M., Larsen, D. S. & van Grondelle, R. Global and Target Analysis of Time-resolved Spectra. *Biochim. Biophys. Acta BBA - Bioenerg.* **1657**, 82–104 (2004).
8. Müller, P., Ignatz, E., Kiontke, S., Brettel, K. & Essen, L.-O. Sub-nanosecond Tryptophan Radical Deprotonation Mediated by a Protein-bound Water Cluster in Class II DNA Photolyases. *Chem. Sci.* **9**, 1200–1212 (2018).
9. Liu, B., Liu, H., Zhong, D. & Lin, C. Searching for a Photocycle of the Cryptochrome Photoreceptors. *Curr. Opin. Plant Biol.* **13**, 578–586 (2010).
10. Langebrake, C. *et al.* Adaptive Evolution and Loss of a Putative Magnetoreceptor in Passerines. *Proc. R. Soc. B Biol. Sci.* **291**, 20232308 (2024).
11. Hoff, A. J. *et al.* The Nuts and Bolts of Distance Determination and Zero- and Double-quantum Coherence in Photoinduced Radical Pairs. *Spectrochim. Acta. A. Mol. Biomol. Spectrosc.* **54**, 2283–2293 (1998).
12. Hochstoeger, T. *et al.* The Biophysical, Molecular, and Anatomical Landscape of Pigeon Cry4: A Candidate Light-based Quantal Magnetosensor. *Sci. Adv.* **6**, eabb9110 (2020).
13. Nohr, D. *et al.* Extended Electron-transfer in Animal Cryptochromes Mediated by a Tetrad of Aromatic Amino Acids. *Biophys. J.* **111**, 301–311 (2016).
14. Bleifuss, G. *et al.* Tryptophan and Tyrosine Radicals in Ribonucleotide Reductase: A Comparative High-field EPR study at 94 GHz. *Biochemistry* **40**, 15362–15368 (2001).
15. Okafuji, A., Schnegg, A., Schleicher, E., Möbius, K. & Weber, S. G-tensors of the Flavin Adenine Dinucleotide Radicals in Glucose Oxidase: A Comparative Multifrequency Electron Paramagnetic Resonance and Electron-nuclear Double Resonance Study. *J. Phys. Chem. B* **112**, 3568–3574 (2008).

16. Zoltowski, B. D. *et al.* Chemical and Structural Analysis of a Photoactive Vertebrate Cryptochrome from Pigeon. *Proc. Natl. Acad. Sci.* **116**, 19449–19457 (2019).
17. Maeda, K. *et al.* Magnetically Sensitive Light-induced Reactions in Cryptochrome are Consistent with its Proposed Role as a Magnetoreceptor. *Proc. Natl. Acad. Sci.* **109**, 4774–4779 (2012).
